# Trajectories of cognitive change following stroke: stepwise decline towards dementia in the elderly

**DOI:** 10.1093/braincomms/fcac129

**Published:** 2022-05-24

**Authors:** João Delgado, Jane Masoli, Yoshiki Hase, Rufus Akinyemi, Clive Ballard, Raj N. Kalaria, Louise M. Allan

**Affiliations:** 1 Epidemiology and Public Health, College of Medicine and Health, University of Exeter, College House, St Lukes, Campus, Exeter EX1 2LT, UK; 2 Healthcare for Older People Department, Royal Devon and Exeter NHS Foundation Trust, RD&E, Barrack Road, Exeter EX2 5D, UK; 3 Translational and Clinical Research Institute, Newcastle University, Campus for Ageing and Vitality, Newcastle upon Tyne NE4 5PL, UK; 4 Institute for Advanced Medical Research and Training, College of Medicine, University of Ibadan, University College Hospital Campus, Ibadan, Nigeria; 5 College of Medicine and Health, University of Exeter, Medical School Building F.04, St Luke's Campus, Exeter EX1 2LU, UK; 6 Centre for Research in Ageing and Cognitive Health, College of Medicine and Health, University of Exeter, South Cloisters 1.40, University of Exeter, St Luke's Campus, Exeter EX1 2LU, UK

**Keywords:** stroke, dementia, cognitive decline, vascular dementia

## Abstract

Stroke events increase the risk of developing dementia, 10% for a first-ever stroke and 30% for recurrent strokes. However, the effects of stroke on global cognition, leading up to dementia, remain poorly understood. We investigated: (i) post-stroke trajectories of cognitive change, (ii) trajectories of cognitive decline in those who develop dementia over periods of follow-up length and (iii) risk factors precipitating the onset of dementia. Prospective cohort of hospital-based stroke survivors in North-East England was followed for up to 12 years. In this study, we included 355 stroke survivors of ≥75 years of age, not demented 3 months post-stroke, who had had annual assessments during follow-up. Global cognition was measured annually and characterized using standardized tests: Cambridge Cognition Examination—Revised and Mini-Mental State Examination. Demographic data and risk factors were recorded at baseline. Mixed-effects models were used to study trajectories in global cognition, and logistic models to test associations between the onset of dementia and key risk factors, adjusted for age and sex. Of the 355 participants, 91 (25.6%) developed dementia during follow-up. The dementia group had a sharper decline in Cambridge Cognition Examination—Revised (coeff. = −1.91, 95% confidence interval = −2.23 to −1.59, *P* < 0.01) and Mini-Mental State Examination (coeff. = −0.46, 95% confidence interval = −0.58 to −0.34, *P* < 0.01) scores during follow-up. Stroke survivors who developed dementia within 3 years after stroke showed a steep decline in global cognition. However, a period of cognitive stability after stroke lasting 3 years was identified for individuals diagnosed with dementia in 4–6 years (coeff. = 0.28, 95% confidence interval = −3.28 to 3.8, *P* = 0.88) of 4 years when diagnosed at 7–9 years (coeff. = −3.00, 95% confidence interval = −6.45 to 0.45, *P* = 0.09); and of 6 years when diagnosed at 10–12 years (coeff. = −6.50, 95% confidence interval = −13.27 to 0.27, *P* = 0.06). These groups then showed a steep decline in Cambridge Cognition Examination—Revised in the 3 years prior to diagnosis of dementia. Risk factors for dementia within 3 years include recurrent stroke (odds ratio = 3.99, 95% confidence interval = 1.30–12.25, *P* = 0.016) and previous disabling stroke, total number of risk factors for dementia (odds ratio = 2.02, 95% confidence interval = 1.26–3.25, *P* = 0.004) and a Cambridge Cognition Examination—Revised score below 80 at baseline (odds ratio = 3.50, 95% confidence interval = 1.29–9.49, *P* = 0.014). Our unique longitudinal study showed cognitive decline following stroke occurs in two stages, a period of cognitive stability followed by rapid decline before a diagnosis of dementia. This pattern suggests stroke may predispose survivors for dementia by diminishing cognitive reserve but with a smaller impact on cognitive function, where cognitive decline may be precipitated by subsequent events, e.g. another cerebrovascular event. This supports the assertion that the development of vascular dementia can be stepwise even when patients have small stroke lesions.

## Teaser

Stroke increases risk of dementia in 30% of survivors. Delgado *et al*.’s unique prospective-longitudinal study showed cognitive decline following stroke occurs in two stages; a period of cognitive stability followed by rapid decline 3 years prior to dementia. We suggest stepwise development of dementia even when patients have small ischaemic lesions.

## Graphical Abstract

**Graphical Abstract:**
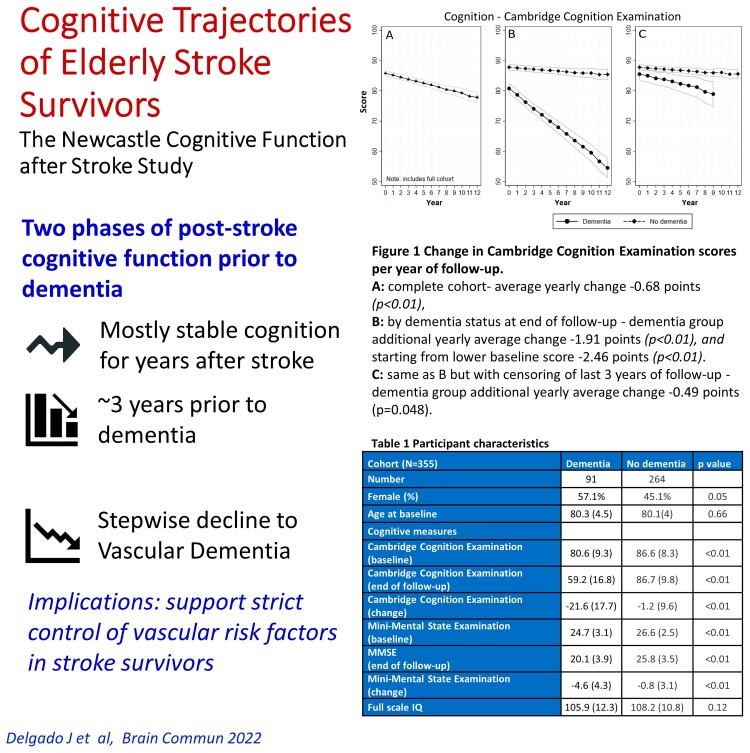

## Introduction

In the UK, over 100 000 cases of stroke occur each year (117 600 in 2015) and this is expected to increase by 60% by 2035 (*n* = 186 900).^1^ Stroke remains a leading cause of death, long-term disability and cognitive impairment.^2,3^ There is an established link between incident stroke and cognitive decline, namely the development of vascular dementia, with around 10% of individuals developing dementia following a first-ever stroke and 30% after recurrent stroke.^2^

Stroke may bring forward a dementia diagnosis by 10 years.^4,5^ Levine *et al*.^6^ demonstrated that an acute decline in cognition at the time of stroke is followed by a persistent linear decline in the following years. However, the mechanisms and the direct effects of stroke on global cognition, as well as on executive function leading up to dementia remain poorly understood,^4^ as is the relationship between brain injury caused by stroke, brain reserve, the ability for the brain to withstand injury and onset of dementia.^5^ Risk factors have been proposed for post-stroke dementia, outside of markers for stroke complications, include age, female sex, low education, race, diabetes and atrial fibrillation which are also known risk factors for Alzheimer’s dementia or pre-stroke dementia.^7,8^ Also, linear trajectories for post-stroke cognitive decline described previously are at odds with the descriptions of cognitive decline for vascular dementia.^6,8^ A linear trajectory describes a progressive decline that is more characteristic of Alzheimer’s dementia, while vascular dementia is ascribed a fluctuating,^9^ or a stepwise cognitive decline towards dementia.^10,11^ This latter trajectory is consistent with findings that multiple and recurrent strokes are predictive of dementia.^8^

There remain significant gaps in our understanding of cognitive decline following a stroke, and how it may in the long-term lead, or describe the progression towards dementia.^8^ We explored cognitive function trajectories in our longitudinal prospective study of elderly stroke survivors.^3^ In the Newcastle cognitive function after stroke (CogFAST) cohort, we previously showed that >75% of stroke survivors develop vascular dementia meeting criteria for severe vascular cognitive impairment per the Vascular Impairment of Cognition Classification Consensus Study consortium criteria.^12^ Here, we investigated: (i) trajectory of cognitive decline in following stroke, in individuals that develop dementia against those who do not, (ii) characteristics of cognitive change in post-stroke survivors who develop dementia and (iii) and risk factors precipitating onset of dementia.^3^

## Materials and methods

The CogFAST cohort is a secondary-care-based longitudinal study of older people (aged 75 year and older) who were diagnosed with stroke in hospital and established by neuroimaging.^3^ Stroke was defined according to the World Health Organization definition and classified according to the Oxford Community Stroke project classification (OCSP).^3^ We focussed on 355 individuals without dementia at baseline (3 months post-stroke) that were followed until diagnosed with dementia or loss to follow-up. For this study, we included up to 12 years of follow-up, the last year of follow-up with a diagnosis of dementia. Participants were separated into to two groups: (i) a ‘dementia’ group includes all individuals who develop dementia at any time during the 12-year follow-up, (ii) a comparison group, named ‘no dementia’ who did not develop dementia during follow-up.

Participants were free from dementia at baseline and from disabilities precluding computer-assisted cognitive testing (e.g. aphasia, hemiparesis affecting the hand used for writing).^13^ Participants received annual clinical and neuropsychological assessments. Cambridge Cognition Examination Revised (CAMCOG-R) and Mini-Mental State Examination (MMSE) scores were calculated for all participants at baseline. CAMCOG-R is a standardized test for global cognitive performance (maximum score of 107), subdivided into 10 domains for memory (27 points), orientation (10 points), language comprehension (9 points), language expression (21 points), attention (7 points), praxis (12 points), calculation (2 points), abstract thinking (8 points) and perception (11 points).^3,14^ A separate executive function domain was scored out of 28 points. However, this new item did not contribute to the total CAMCOG-R score. MMSE provides a quantitative estimate of severity of cognitive impairment’ (maximum of 30 points), based on questions covering seven domains: orientation to time (5 points); orientation to place (5 points); registration of three words (3 points); attention and calculation (5 points); recall of three words (3 points); language (8 points) and visual construction (1 point). The items for the MMSE in this study were embedded in the CAMCOG-R assessment. IQ at baseline was assessed using the National Adult Reading Test.^15^ However, since the MMSE is widely used, we also computed MMSE scores separately for all the subjects to assess their utility in comparison with the more comprehensive CAMCOG battery. Currently used criteria for mild cognitive impairment were not incorporated in the quantitative measures but we did determine individuals who had cognitive impairment no dementia^4,16^ equivalent to mild vascular cognitive impairment.^12^

### Statistical analysis

The statistical plan presented in this manuscript was developed for CAMCOG-R and then repeated in its entirety for MMSE on the same sample.

#### Trajectories of cognitive change

Trajectories of cognitive function (measured by the CAMCOG-R and MMSE) during the follow-up period were modelled as a function of the years from baseline until an individual’s end of follow-up. We used random linear mixed-effects models, fitted via maximum likelihood with random effects specified at individual level. Analysis also included adjustments for age (<80 and ≥80 years) and sex.^17^ Three linear models (L) were produced: (L1) full cohort for full duration of the follow-up; (L2) full cohort with an interaction term for diagnosis of dementia at end of follow-up and (L3) full cohort with an interaction term for dementia and censoring of the last years of follow-up. The latter analysis, censoring the last 3 years of cognitive measures, investigates whether cognitive change follows a stepwise or continuous pattern. In continuous change over time censoring, the last years of follow-up should have a minimal effect on yearly rate of change, but a marked difference if the pattern is stepwise.

For the dementia group, we produced analysis stratifying participants into four groups by length of follow-up, which includes individuals with up to 1–3, 4–6, 7–9 and 10–12 years of follow-up time. These were named as 0–3Y, 4–6Y, 7–8Y and 10–12Y, respectively. We used random linear mixed-effects models, with follow-up years as indicator variables to account for the nonlinear form of trajectories [hereafter referred to as the step-function (SF) model]. This implementation compares each year of follow-up against a baseline value, allowing for identifying possible inflection points that are concealed by linear models. Two sets of SF models were produced: (SF1) complete follow-up and (SF2) censoring the last 3 years of follow-up.

Results from mixed-effects models are displayed as profile plots, produced using population marginal means or predicted means.^17^ These are estimated from the fitted model and preferred to observed means which do not account for the underlying model of the data.^18,19^ Profile plots are useful for comparing marginal means in the model, where a line plot in which each point indicates the estimated marginal mean of a dependent variable (adjusted for covariates) at one level of a factor.

#### Declines in CAMCOG during follow-up

For people with dementia, we analysed where within the follow-up time, large reductions in CAMCOG score from 1 year of follow to the next were more common. We estimated the proportion of individuals who had a decline in CAMCOG score of (i) at least 5 points and (ii) at least 10 points at 4 stages of follow-up starting in the 3, 4–6, 7–8 and 10–12 years before diagnosis. Analyses were performed separately for each follow-up category for the 0–3Y, 4–6Y, 7–8Y and 10–12Y groups.

### Risk factors of step cognitive decline

We analysed risk factors for early onset of cognitive decline, defined as the group of participants that developed dementia within 3 years after stroke, compared with those that developed after 3 or more years. Risk factors include: OCSP classification, cognitive impairment with no dementia (CIND) or mild vascular cognitive impairment,^12^ previous stroke, previous disabling stroke, apolipoprotein E (*APOE*) ε4, hypertension, myocardial infarction, ischaemic heart disease, type 2 diabetes, atrial fibrillation, hypercholesterolaemia, smoking history and number of risk factors (SD). We used logistic regression models adjusted for age and sex. Univariate analysis of risk factors was adjusted for continuous age and gender. Multivariate analysis included all risk factors in addition to age and sex, except for number of risk factors to avoid possible over adjustment. Previous disabling stroke was not included in either analysis as 0 case was detected in the group who developed dementia after 3 or more years.

Analyses were performed using STATA version 15, 2017. Stata Statistical Software: Release 15. StataCorp LLC. For all analyses, statistical significance was set at *P* < 0.05.

### Data availability

The data that support the findings of this study are available on request from the corresponding or senior authors. The data are not publicly available due to privacy or ethical restrictions.

## Results

Of the *n* = 355 participants enrolled in the CogFAST study between 1999 and 2003, *n* = 91 (25.6%) individuals developed dementia during follow-up (Table 1). The two groups were comparable in age (*P* = 0.66) and sex (*P* = 0.05), stroke types (*P* = 0.83), with predominantly ischaemic infarction in both groups, and IQ (*P* = 0.12). The dementia group had lower CAMCOG-R scores at baseline (*P* *<* 0.01) although not diagnostic for dementia and at the end of follow-up (*P* < 0.01). The dementia group was followed for a total of 310 person-years (mean = 3.4, SD = 2.6) and the non-dementia group for a total of 900 person-years (mean = 3.4, SD = 3.3).

**Table 1:** Demographic characteristics

Variable	Dementia	No dementia	*P*-value
Number	91	264	
Female (%)	52 (57.1)	119 (45.1)	0.05
Age at baseline	80.3 (4.5)	80.1 (4)	0.66
Final diagnosis	–	–	
Ischaemic infarction	83 (91.2)	228 (86.4)	0.828
Haemorrhagic infarction	2 (2.2)	4 (1.5)	–
Intracerebral haemorrhage	2 (2.2)	9 (3.4)	–
TIA	1 (1.1)	7 (2.7)	–
Multiple	0 (0)	1 (0.4)	–
Not known	0 (0)	2 (0.8)	–
Missing	3 (3.3)	13 (4.9)	–
*Cognitive measures*			
CAMCOG-R (baseline)	80.6 (9.3)	86.6 (8.3)	<0.01
CAMCOG-R (*Z*-score baseline)	−0.49 (1.0)	0.17 (0.93)	<0.01
CAMCOG-R (end of follow-up)	59.2 (16.8)	86.7 (9.8)	<0.01
CAMCOG-R (*Z*-score end of follow-up)	−1.0 (0.9)	0.4 (0.7)	<0.01
Change in CAMCOG-R	21.6 (17.7)	1.2 (9.6)	<0.01
MMSE (baseline)	24.7 (3.1)	26.6 (2.5)	<0.01
MMSE *Z*-score (*Z*-score baseline)	−0.50 (1.1)	0.17 (0.9)	<0.01
MMSE (end of follow-up)	20.1 (3.9)	25.8 (3.5)	<0.01
MMSE (*Z*-score end of follow-up)	−0.9 (0.9)	0.3 (0.8)	<0.01
Change in MMSE	−4.6 (4.3)	−0.8 (3.1)	<0.01
Full scale IQ	105.9 (12.3)	108.2 (10.8)	0.12
Verbal IQ	104.6 (11.3)	106.7 (10)	0.12
*Follow-up in years*	–	–	–
Baseline only	0 (0.0)	58 (22.0)	<0.01
1–3	61 (67.0)	105 (39.8)	–
4–6	19 (20.9)	47 (17.8)	–
7–9	7 (7.7)	41 (15.5)	–
10–12	4 (4.4)	13 (4.9)	–
*OCSP classification*	–	–	–
LACS	29 (31.9)	87 (33.0)	0.78
PACS	38 (41.8)	107 (40.5)	–
TACS	7 (7.7)	12 (4.5)	–
POCS	10 (11.0)	39 (14.8)	–
Not classified	7 (7.7)	19 (7.2)	
CIND (CAMCOG-R <80)	31 (50.8)	7 (23.3)	–

### Change in cognition after stroke: linear model

**Figure 1:**
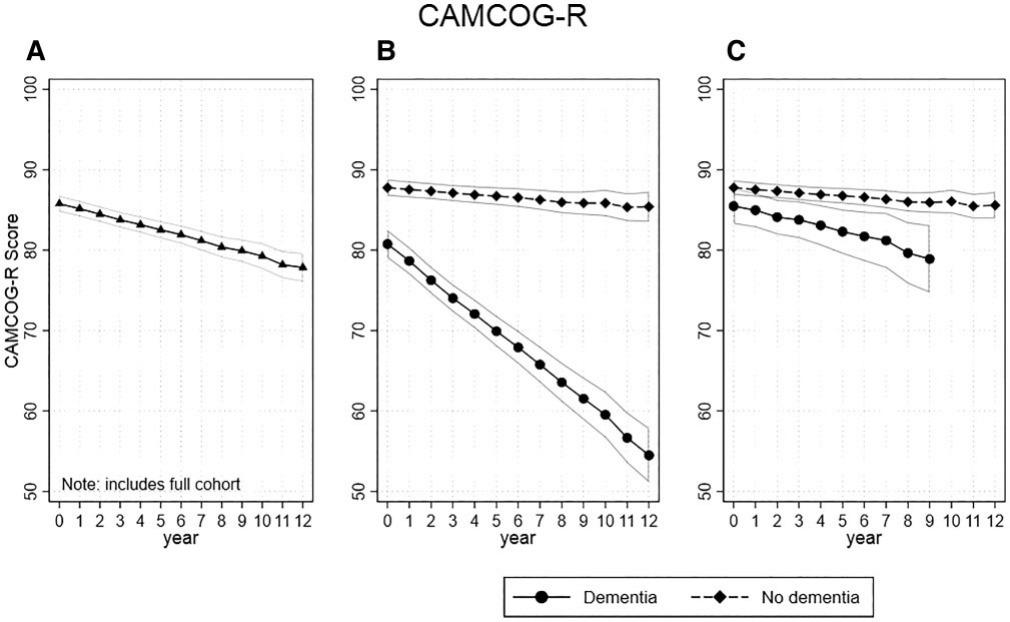
**Trajectory of CAMCOG scores per year of follow-up.** (**A**) Complete cohort changed an average of *0.68* points per year. (**B**) By dementia status at end of follow-up, CAMCOG scores in the dementia group compared with the no-dementia group (coeff. = −1.91, *P* < 0.01) and with the dementia groups starting from a lower score at baseline (coeff. = −2.46, *P* < 0.01). (**C**) Same as **B** but with censoring of last 3 years of follow-up (coeff. = −0.49, *P* = 0.048).

**Figure 2:**
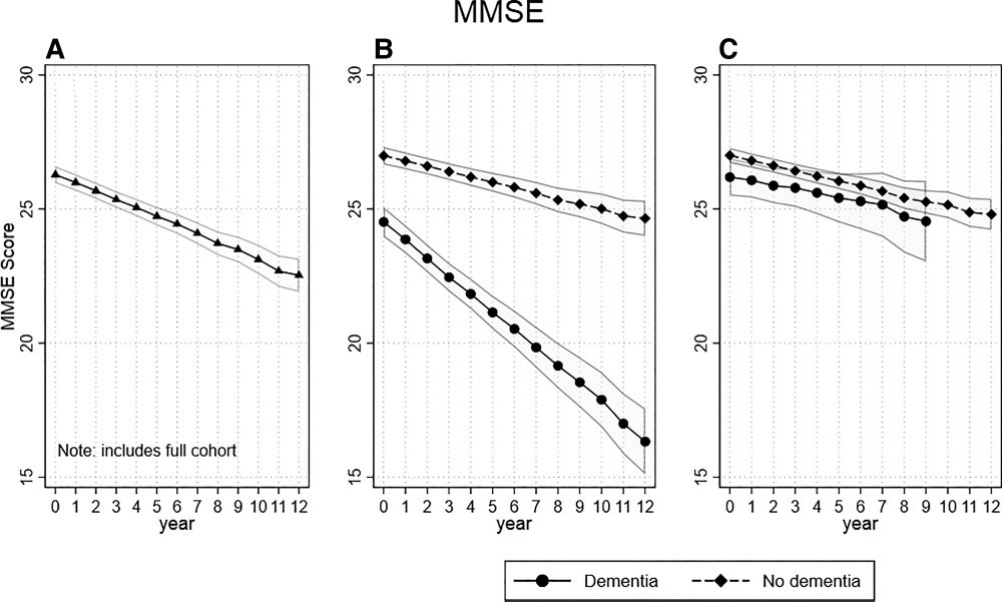
**Trajectory of MMSE score per year of follow-up.** (**A**) Complete cohort, MMSE showed a yearly decline of −0.32. (**B**) By dementia status at end of follow-up, the dementia group had a steeper yearly decline (coeff. = −0.46, *P* < 0.01), as well as a lower score at baseline (coeff. = −2.46, *P* < 0.01). (**C**) Same as **B** but with censoring of last 3 years of follow-up (coeff. = 0.03, *P* = 0.788).

CAMCOG changed an average of 0.68 [95% confidence interval (CI) = −0.81 to −0.54, *P* < 0.01) points per year of follow-up in the L1 models (Fig. 1A and Supplementary Table 1). The L2 model with interaction terms for dementia identified a steeper yearly decline in CAMCOG scores in the dementia group compared with the no-dementia group (coeff. = −1.91, 95% CI = −2.23 to −1.59, *P* < 0.01) with the dementia groups starting from lower score at baseline (coeff. = −2.46, 95% CI = −3.11 to −1.81, *P* < 0.01; Fig. 1B). The L1 model applied to MMSE found a similar trend with combined groups showing a yearly decline of −0.32 (95% CI = −0.37 to −0.26, *P* < 0.01; Fig. 2A and Supplementary Table 2), while the L2 model with interaction term also found the dementia group had a steeper yearly decline (coeff. = −0.46, 95% CI = −0.58 to −0.34, *P* < 0.01), as well as a lower score at baseline (coeff. = −2.46, 95% CI = −3.11 to −1.81, *P* < 0.01; Fig. 2B and Supplementary Table 2).

### Change in cognition in the dementia group

**Figure 3:**
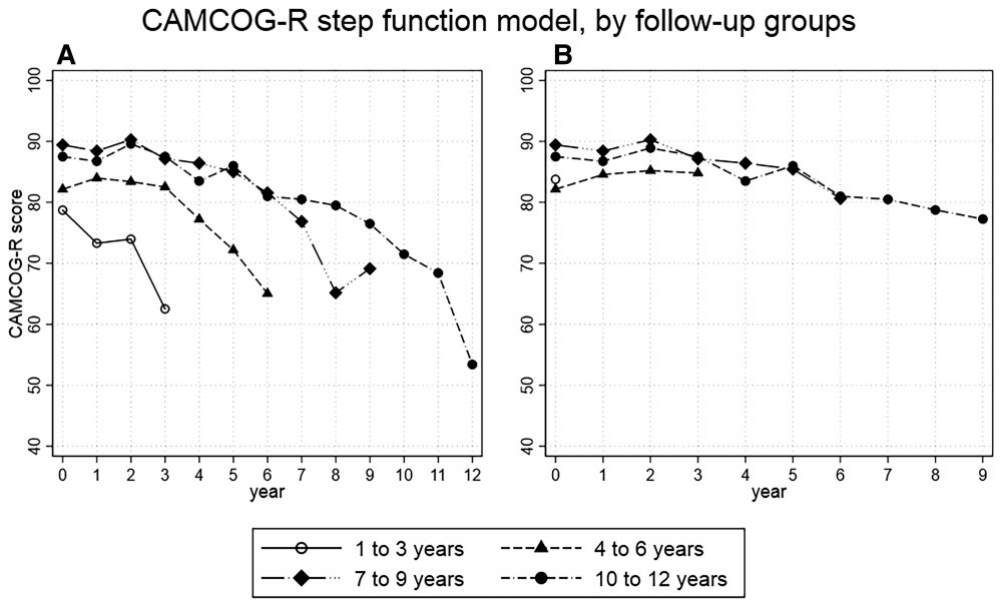
**CAMCOG score per year of follow-up in the incident dementia group.** (**A**) Stratified by length of follow-up, in 3-year segments. (**B**) Same as **A** but with censoring of last 3 years of follow-up. Trajectories of cognitive change remain mostly stable throughout the follow-up period with little decline at the end of follow-up (4–6 years: coeff. = 2.77, *P* = 0.18; 7–9 years: coeff. = −8.43, *P* < 0.01; 10–12 years: coeff. = −9.56, *P* < 0.01).

**Figure 4:**
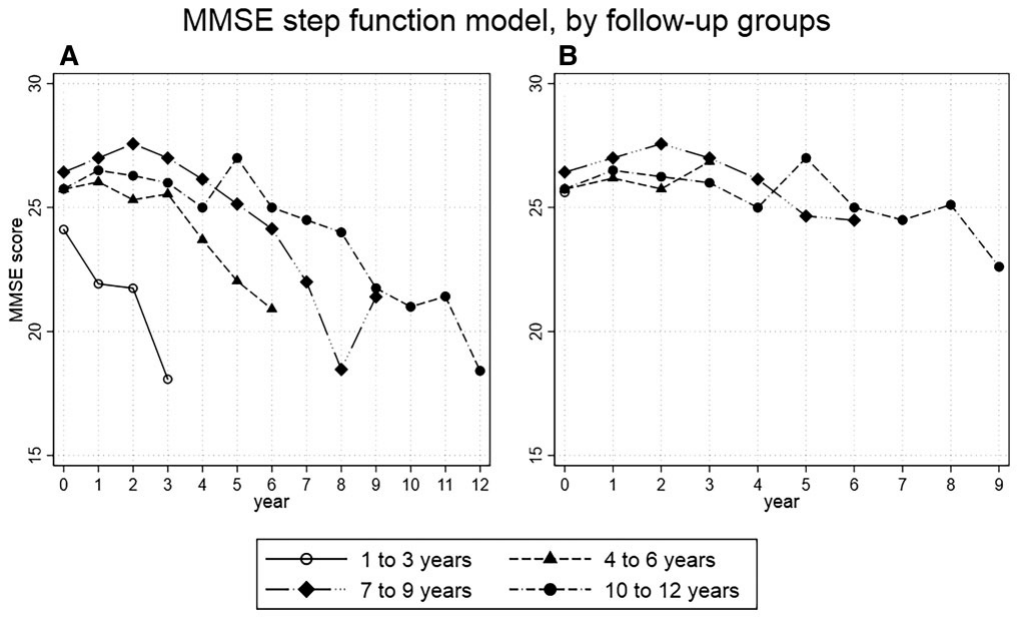
**MMSE score per year of follow-up in the incident dementia group.** (**A**) Stratified by length of follow-up, in 3 year segments. (**B**) Same as **A** but with censoring of last 3 years of follow-up. MMSE showed similar results (4–6 years: coeff. = 1.04, *P* < 0.083; 7–9 years: coeff. = −1.78, *P* < 0.160; 10–12 years: coeff. = −3.00, *P* < 0.025).

The SF1 model describes CAMCOG trajectories, stratified by length of follow-up (Fig. 3A). All four groups showed a decline in CAMCOG over the full follow-up period (1–3Y coeff. = −15.24, 95% CI = −19.78 to −10.70, *P* < 0.01; 4–6Y coeff. = −17.43, 95% CI = −22.29 to −12.57, *P* < 0.01; 7–9Y coeff. = −20.21, 95% CI = −25.54 to −14.88, *P* < 0.01; 10–12Y coeff. = −33.49, 95% CI = −41.91 to −25.07, *P* < 0.01; Supplementary Table 3). The 1–3Y group demonstrated stepwise decline in CAMCOG score over the 3 years of follow-up (Fig. 3A). However, groups with longer follow-ups showed a period of cognitive stability where CAMCOG was not statistically different from baseline, lasting 3 years for the 4–6Y (coeff. = 0.28, 95% CI = −3.28 to 3.8, *P* = 0.88); 4 years for the 7–9Y (coeff. = −3.00, 95% CI = −6.45 to 0.45, *P* = 0.09) and 6 years for the 10–12Y (coeff. = −6.50, 95% CI = −13.27 to 0.27, *P* = 0.06). This was followed by a steep decline in the last 3 years of follow-up (Fig. 3A and Supplementary Table 5). Application of the SF1 model to MMSE produced similar results, with a period of cognitive stability of 3 years 4–6Y (coeff. = −0.19, 95% CI = −1.44 to 1.06, *P* = 0.764), 5 years for the 7–9Y (coeff. = −1.29, 95% CI = −3.23 to 0.66, *P* = 0.196), 8 years for the 10–12Y (coeff. = −1.75, 95% CI = −4.30 to 0.80, *P* = 0.178), with cognitive decline occurring in the last 3 years of follow-up (Fig. 4A and Supplementary Table 7).

Decline was also observed in all individual CAMCOG components in the last 3 years of follow-up (Table 2 and Supplementary Fig. 1). The greatest yearly declines were observed for orientation with a −7.06% (95% CI = −8.62 to −5.51, *P* < 0.001), −6.04% for total memory (95% CI = −7.14 to −4.95, *P* < 0.001), −4.52% for attention (95% CI = −6.37 to −2.67, *P* < 0.001) and −4.09% for praxis (95% CI = −5.49 to −2.69, *P* < 0.001).

**Table 2:** Change in CAMCOG component in the last 3 year of follow in the dementia group

		Total score				Percentage change			
	Max	Coeff.	LL	UL	*P*-value	%	LL	UL	*P*-value
CAMCOG-R	107	−4.6	−5.5	−3.8	0.00	−4.4	−5.1	−3.6	0.00
Orientation	10	−0.7	−0.9	−0.6	0.00	−7.1	−8.6	−5.5	0.00
Memory total	27	−1.6	−1.9	−1.3	0.00	−6.0	−7.1	−5.0	0.00
Language total	30	−0.7	−0.9	−0.5	0.00	−2.3	−3.0	−1.5	0.00
Attention	9	−0.4	−0.6	−0.2	0.00	−4.5	−6.4	−2.7	0.00
Praxis	12	−0.5	−0.7	−0.3	0.00	−4.1	−5.5	−2.7	0.00
Perception	10	−0.3	−0.4	−0.1	0.00	−2.8	−4.2	−1.4	0.00
Abstract thinking	8	−0.2	−0.3	0.0	0.08	−2.0	−4.2	0.3	0.08
Total executive^a^	28	−0.7	−1.1	−0.4	0.00	−2.6	−3.8	−1.5	0.00

^a^ Does not contrite to total CAMCOG-R score.

### Follow-up with last 3 years censored

The SF2 model, with censoring of the least 3 years of follow-up, showed trajectories of cognitive change remain mostly stable throughout the follow-up period with little decline at the end of follow-up (4–6Y: coeff. = 2.77, 95% CI = 1.30–6.85, *P* = 0.18; 7–9Y: coeff. = −8.43, 95% CI = −12.28 to −4.58, *P* < 0.01; 10–12Y: coeff. = −9.56, 95% CI = −16.63 to −2.49, *P* < 0.01; Supplementary Table 6 and Fig. 3B). The SF2 model applied to MMSE showed similar results (4–6Y: coeff. = 1.04, 95% CI = −0.13 to 2.21, *P* < 0.083; 7–9Y: coeff. = −1.78, 95% CI = −4.26 to 0.70, *P* < 0.160; 10–12Y coeff. = −3.00, 95% CI = −5.62 to −0.37, *P* < 0.025; Fig. 4B and Supplementary Table 8). The 1–3Y group was excluded from the SF2 model as no observations were available after censoring.

The L3 models, with interaction term and with censoring of the last 3 years of follow-up for the dementia group, showed trajectories of cognitive change were only marginally different between the dementia and no-dementia group for CAMCOG (coeff. = −0.49, 95% CI = −0.97 to 0.00, *P* = 0.048; Supplementary Table 1) and not statistically different for MMSE (coeff. = 0.03, 95% CI = −0.16 to 0.21, *P* = 0.788; Supplementary Table 2).

### Precipitating factors for onset of dementia in first 3 years

Univariate models produced to identify risk of developing dementia within 3 years of follow-up identified history of recurrent stroke was associated with increased risk of developing dementia within 3 years after the event [odds ratio (OR) = 3.99, 95% CI = 1.30–12.25, *P* = 0.016]. All individuals with a history of previously disabling stroke developed dementia within 3 years, indicating an infinite OR (Table 3). Likelihood of diagnosis of dementia increased with increasing number of risk factors (OR = 2.02, 95% CI = 1.26–3.25, *P* = 0.004), although a stratified analysis on number of risk factors was not significant (Table 3). CIND at baseline was also associated with increased risk of dementia within 3 years (OR = 3.50, 95% CI = 1.29–9.49, *P* = 0.014). No other risk factors were statistically significant, although few trends were noteworthy including type 2 diabetes (OR = 4.50, 95% CI = 0.53–38.36, *P* = 0.169), hypercholesterolaemia (OR = 3.44, 95% CI = 0.38–31.56, *P* = 0.274) and hypertension (OR = 2.31, 95% CI = 0.92–5.81, *P* < 0.074; Table 3).

**Table 3:** Univariate and multivariate predictors of death

	Dementia in		Univariate model				Multivariate model			
	≤ 3 years	> 3 years	OR	LL	UL	*P*-value	OR	LL	UL	*P*-value
Number	–	–	–	–	–	–	–	–	–	–
OCSP classification	–	–	–	–	–	–	–	–	–	–
LACS	19 (31.1)	10 (33.3)	Ref.	–	–	–	–	–	–	–
PACS	26 (42.6)	12 (40.0)	1.15	0.41	3.25	0.789	0.77	0.23	2.57	0.665
TACS	6 (9.8)	1 (3.3)	3.18	0.33	30.55	0.317	4.61	0.34	62.01	0.249
POCS	5 (8.2)	5 (16.7)	0.48	0.11	2.15	0.339	0.26	0.04	1.78	0.168
Number of risk factors (SD)^a^	–	–	–	–	–	–	–	–	–	–
0	3 (10.0)	6 (9.8)	Ref.	–	–	–	–	–	–	–
1	15 (50.0)	12 (19.7)	0.38	0.08	1.90	0.240	–	–	–	–
2	9 (30.0)	20 (32.8)	1.07	0.22	5.33	0.933	–	–	–	–
3 or more	3 (10.0)	23 (37.7)	3.80	0.60	23.88	0.155	–	–	–	–
Count of risk factors	–	–	2.02	1.26	3.25	0.004				
Previous stroke	25 (41.0)	5 (16.7)	3.99	1.30	12.25	0.016	3.74	0.91	15.39	0.068
Previous disabling stroke^b^	15 (24.6)	0 (0.0)	–	–	–	–	–	–	–	–
CIND	31 (50.8)	7 (23.3)	3.50	1.29	9.49	0.014	3.00	0.86	10.40	0.084
APOE ε4	13 (21.3)	9 (30.0)	0.60	0.22	1.65	0.320	1.15	0.30	4.39	0.843
Hypertension	39 (63.9)	14 (46.7)	2.31	0.92	5.81	0.074	3.07	0.91	10.39	0.072
Myocardial infarction	14 (23.0)	6 (20.0)	1.32	0.43	4.00	0.628	1.14	0.28	4.63	0.855
Ischaemic heart disease	25 (41.0)	11 (36.7)	1.29	0.52	3.20	0.583	1.14	0.32	4.08	0.846
Type 2 diabetes	8 (13.1)	1 (3.3)	4.50	0.53	38.36	0.169	6.14	0.36	105.23	0.211
Atrial fibrillation	11 (18.0)	4 (13.3)	1.50	0.43	5.18	0.525	1.34	0.22	8.03	0.746
Hypercholesterolaemia	6 (9.8)	1 (3.3)	3.44	0.38	31.56	0.274	1.00			
Smoking history	38 (62.3)	18 (60.0)	1.08	0.41	2.85	0.878	1.04	0.26	4.08	0.958
Age	–	–	0.98	0.89	1.08	0.66	0.95	0.83	1.09	0.499
Sex	–	–	0.97	0.40	2.35	0.95	1.36	0.39	4.78	0.631

^a^ Excluded from multivariate model due to possible over adjustment.

^b^ Excluded as not cases were identified in >3-year dementia group.

Lastly, compared with lacunar stroke, partial anterior circulation stroke (PACS) show a non-significant association with dementia within 3 years (OR = 1.15, 95% CI = 0.41–3.25, *P* = 0.789) and total anterior circulation stroke syndrome (TACS) showed a stronger relationship still (OR = 3.18, 95% CI = 0.33–30.55, *P* = 0.317), while those with posterior circulation stroke (POCS) seemed to indicate reduced risk of dementia within 3 years (OR = 0.48, 95% CI = 0.11–2.15, *P* = 0.339). The multivariate analysis attenuated most ORs with none of the candidate risk factors achieving significance. *APOE* ε4 was not associated with a diagnosis of dementia within 3 years (Table 3).

## Discussion

Our unique, large study in older (≥75 years age) stroke survivors in the North-East of England characterized the relationship between trajectories of cognitive function and post-stroke dementia during up to 12 years of follow-up in a hospital-based cohort of survivors of first or recurrent stroke. It showed that decline in global cognition following stroke follows a pattern of cognitive decline that can be more precisely described as fluctuating,^9^ or stepwise.^10,11^ Such a pattern is traditionally associated with VaD caused by multiple infarcts. Our findings on the incidence of dementia of 26% are also generally in agreement with those from the Oxford Vascular Study reporting 34% incidence of post-event dementia at 1 year, particularly in patients with severe stroke.^20,21^ Interestingly, these incidence rates are not very different from those in Nigerian stroke survivors with different dietary and lifestyle factors.^21^

In the dementia group, the SF1 model showed a steep linear decline in global cognition for those developing dementia within the first 3 years of follow-up. However, individuals with longer follow-up had a period of cognitive stability immediately after stroke which could last up to 8 years, with steep declines starting only in the 3 years before a dementia diagnosis. After excluding the last 3 years of follow-up in the dementia group (SF2 model), these individuals showed similar, mostly flat trajectories of cognitive function regardless of follow-up time. Most importantly, in the linear models where the dementia groups showed a steeper decline global cognition compared with the no dementia for CAMCOG-R and MMSE (L2 model), after excluding the last 3 years of follow-up, the cognitive trajectory from the dementia group was not dissimilar, or only modestly dissimilar from that of the no-dementia group. These findings contradict previous characterizations of progressive but persistent cognitive decline.^6^ They also suggest these individuals may not yet have been on a path towards dementia, and thus while stroke may predispose for dementia, cognitive decline may only occur after a subsequent precipitating event, i.e. a further assault or injury to the brain.

Data on incident stroke and other potential causes for brain injury were not collected during follow-up and therefore, we cannot identify whether subsequent milder or covert events, which may have not required hospitalization precipitated cognitive decline. Nonetheless, a risk factor analysis for developing dementia within 3 years of stroke (versus >3 years) found a history of previous stroke at baseline was a precipitating factor for dementia, which is consistent with previous findings.^3^ Severity of stroke was also a precipitating factor. For example, all individuals with a record of disabling stroke developed dementia within 3 years, while stroke types, based OCSP classification displayed a dose–response trend where strokes causing the greatest damage to the frontal-temporal area were more likely to result in dementia within 3 years.^8^ Remarkably, the presence of the *APOE* ε4 was not associated with the onset of dementia in 3 years.^22^ This appears consistent that post-stroke survivors develop VaD and lack Alzheimer type of pathology or amyloid burden as determined by Pittsburgh compound-B binding in >70% of post-stroke survivors.^3,23^ We previously reported that >75% of the post-mortem cases fit into VaD criteria.^3,12,24^ Our subsequent analyses and pathological findings suggest even more cases can be classed as VaD. The rest of the cases were mixed with diagnostic AD type of pathology (Kalaria *et al*., unpublished data).

Our findings are in accord with the concepts of both cognitive and brain reserve.^25^ In the biological sense of brain reserve, the brain in the stroke survivors likely sustains a certain level of stroke injury before clinical or cognitive deficit emerges.^5,26^ However, cognitive reserve may also play a role in sustaining the severity of injury, and the degree of cumulative lesion burden as factors in cognitive outcomes.^5^ Our findings show those diagnosed with dementia within 3 years had evidence of previous brain injury, i.e. previous stroke, or suffered from an overt stroke, causing depletion of brain reserve and cognitive decline. In contrast, those diagnosed with dementia after >3 years were more likely to have milder forms of stroke. This concept and our findings suggest that individuals with previous but not debilitating stroke have a predisposition to cognitive decline, where the brain can function normally but in a state of heightened susceptibility to additional injury. In cases where additional injury occurs, it can precipitate relatively rapid cognitive decline towards dementia. Clinically, this has implications for both the follow-up of people who have had a stroke and for history taking in memory clinic settings. Primary care physicians should be aware that those who appear to be stable after a stroke may subsequently go on to develop further episodes of stroke or brain injury and they need to enquire about cognitive step down at routine annual reviews of these patients. In the memory clinic setting, it is useful to enquire about the pattern of cognitive decline after stroke and be aware that further brain events can cause a step down in cognition which point to a vascular cause of dementia. Clinicians should ask about further stroke symptoms even if these are transient or covert events possibly uncovered by re-imaging.^27^ Furthermore, controlling vascular risk factors in these patients should be vigorously pursued.^3^

Examination of cognitive domains showed that trajectories were steeper in orientation, memory, attention and praxis. It is not surprising that memory was particularly affected as we used a definition of dementia which includes change in memory as a criterion. However, the changes in attention and praxis may be domains which the clinician could particularly look out for during follow-up. It is not unlikely that deficits in these cognitive domains reflect the progressive disruption of the fronto-subcortical circuits also suggested by aberrations in the default mode network.^28^ Both orientation or spatial navigation neglect and memory may be associated with the disconnection with the temporal lobe, in particular, the hippocampal formation.^29^

### Strengths and limitations

Our distinctive study provides a longitudinal analysis of change in global cognition following stroke and followed individuals. It includes a sample of *n* = 355 participants who were followed for up to 12 years, the longest follow-up available in a cohort study focusing on post-stroke dementia. Cognitive function was measured with two standardized and peer-reviewed methods the CAMCOG-R and the MMSE score. Analysis of trajectories of cognitive decline produced in L and SF models were replicated across both measures of cognitive decline.

There are few limitations to this study. We found only 91 of the 355 participants were diagnosed with dementia. This impacts analysis focusing on a limited number of survival groups. Ideally, in SF1 and SF2, analysis should have included 12 groups, stratifying by each year follow-up duration. However, this was not possible due to sample size limitations. The compromise solution was stratifying participants into four groups which still allowed detection of differences by length of follow-up time. This also impacted the available statistical power to identify risk factors for diagnosis of dementia within 3 years. Survivorship bias is a possible limitation, as some participants in the non-dementia group may have been lost to follow-up before developing dementia. This is unlikely to have affected the analysis of trajectories where the non-dementia group sustained cognitive function throughout follow-up, or the analysis of risk factors which we limited to the dementia group. Lastly, it was not possible within the resources of the study to establish, with accuracy, whether further strokes by neuroimaging and other events to detect further vascular brain injury, had occurred during follow-up.

## Conclusion

This unique large study in older (≥75 years age) stroke survivors produced evidence that older people who experience stroke can have a period of stable cognitive function for a number of years after stroke. However, the notable finding is that post-stroke survivors undergo a remarkable decline ∼3 years before the dementia threshold. Collectively, this can then be followed by a stepwise decline which should alert the clinician to a possibility of impending VaD. These findings have implications for life after stroke and patient follow-up in stroke and memory clinics.

## Supplementary Material

fcac129_Supplementary_Data

## Abbreviations

APOE =: apolipoprotein E
CAMCOG-R =: Cambridge Cognition Examination—Revised
CIND =: cognitive impairment with no dementia
CogFAST =: Newcastle cognitive function after stroke
L =: linear model
MMSE =: Mini-Mental State Examination
OCSP =: Oxford Community Stroke project classification
PACS =: partial anterior circulation stroke
POCS =: posterior circulation stroke
SF models =: step-function models
TACS =: total anterior circulation stroke syndrome
