# Supplementary material for: Trajectories of cognitive change following stroke: stepwise decline towards dementia in the elderly
Source: Brain Commun. 2022 May 24;4(3):fcac129. doi: 10.1093/braincomms/fcac129 (PMC9161377; doi:10.1093/braincomms/fcac129)

OXFORD UNIVERSITY PRESS | Brain Communications

Trajectories of cognitive change following stroke: stepwise decline towards dementia in the elderly

Journal:	*Brain Communications*
Manuscript ID	BRAINCOM-2021-399
Manuscript Type:	Original Article
Date Submitted by the Author:	08-Nov-2021
Complete List of Authors:	Delgado, Joao; University of Exeter,<br>Masoli, Jane; University of Exeter<br>Hase, Yoshiki ; Newcastle University, Centre for Vitality and Ageing<br>Akinyemi, Rufus; Newcastle University, Institute for Ageing and Health<br>Ballard, Clive; University of Exeter, College of Medicine and Health<br>Kalaria, Rajesh; Newcastle University, Centre for Vitality and Ageing<br>Allan, Louise; University of Exeter, Institute of Health Research
Keywords:	

SCHOLARONE™
Manuscripts

Trajectories of cognitive change following stroke: stepwise decline towards dementia in the elderly

Joao Delgado[1], Jane Masoli[1,2], Yoshiki Hase[3], Rufus Akinyemi[3,4], Clive Ballard[5], Raj N Kalaria[3,4†] Louise M Allan[6†],

[†]**Joint senior authors**

For Review Only

Abstract

Stroke events increase the risk of developing dementia, 10% for a first-ever stroke, and 30% for recurrent strokes. However, the effects of stroke on global cognition, leading up to dementia remain poorly understood. We investigated: 1) post-stroke trajectories of cognitive change, 2) trajectories of cognitive decline in those that develop dementia over periods of follow-up length, and 3) risk factors precipitating the onset of dementia.

Prospective cohort of hospital-based stroke survivors in the North East of England was followed for up to 12 years. In this study, we included 355 stroke survivors of ≥75 years of age, not demented 3 months post-stroke, who had had annual assessments during follow-up.

Global cognition was measured at annual study visits and characterised using the standardised tests CAMCOG-R and MMSE indexes. Demographic data and risk factors were recorded at baseline. Mixed effect models were used to study trajectories in global cognition, and logistic models to test associations between the onset of dementia and key risk factors, adjusted for age and sex.

Of the 355 participants 91 (25.6%) developed dementia during follow-up. The dementia group had a sharper decline in CAMCOG-R (coef.=-1.91, CI95%=-2.23:-1.59, p<0.01) and MMSE (coef.=-0.46, CI95%=-0.58:-0.34, p<0.01) scores during follow-up.

Stroke survivors that developed dementia within 3 years after stroke showed a steep decline in global cognition. However, a period of cognitive stability after stroke lasting 3 years was identified for individuals diagnosed with dementia in 4-6 years (coef.=0.28, CI95%=-3.28:3.8, p=0.88) of 4 years when diagnosed at 7-9 years (coef.=-3.00, CI95%=-6.45:0.45, p=0.09; and of 6 years when diagnosed at 10-12 years (coef.=-6.50, CI95%=-13.27:0.27, p=0.06). These

groups then showed a steep decline in CAMCOG-R in the 3 years prior to diagnosis of dementia.

Risk factors for dementia within 3 years include recurrent stroke (OR=3.99 CI95%=1.30:12.25, p=0.016) and previous disabling stroke, total number of risk factors for dementia (OR=2.02 CI95%=1.26:3.25, p=0.004), and a CAMCOG-R score below 80 at baseline (OR=3.50 CI95%=1.29:9.49, p=0.014).

Our unique longitudinal study showed cognitive decline following stroke occurs in two stages, a period of cognitive stability followed by rapid decline before a diagnosis of dementia. This pattern suggests stroke may predispose survivors for dementia by diminishing cognitive reserve but with a smaller impact on cognitive function, where cognitive decline may be precipitated by subsequent events, e.g. another cerebrovascular event. This supports the assertion that the development of vascular dementia can be stepwise even when patients have small stroke lesions.

For Review Only

Author affiliations:

1 Epidemiology and Public Health, College of Medicine and Health, University of Exeter Address: College House, St. Lukes, Campus, 79 Heavitree Rd, Exeter EX1 2LT, UK.

2 Healthcare for Older People Department, Royal Devon and Exeter NHS Foundation Trust Address: RD&E, Barrack Road, Exeter, EX2 5D, UK.

3 Translational and Clinical Research Institute, Newcastle University Address: Campus for Ageing and Vitality, Newcastle upon Tyne, NE4 5PL, UK.

4 Institute for Advanced Medical Research and Training, College of Medicine, University of Ibadan, University College Hospital Campus, Ibadan, Nigeria.

5 College of Medicine and Health, University of Exeter Address: Medical School Building F.04, St Luke's Campus, Magdalen Road, Exeter, EX1 2LU, UK

6 Centre for Research in Ageing and Cognitive Health, College of Medicine and Health, University of Exeter Address: South Cloisters 1.40, University of Exeter, St Luke's Campus, Heavitree Road, Exeter, EX1 2LU, UK

Correspondence to: João Delgado

Full address: University of Exeter, College House 1.02, St. Lukes, Campus, 79 Heavitree Rd, Exeter EX1 2LT

E-mail: j.correa-delgado@exeter.ac.uk

Running title: Trajectories after stroke in the elderly

Keywords: stroke; dementia, cognitive decline; vascular dementia

Abbreviations: APOE = Apolipoprotein E; CAMCOG-R = Cambridge Cognition Examination – Revised; CIND = Cognitive Impairment with No Dementia; CogFAST = Newcastle Cognitive Function after Stroke; CAMCOG-R = The Cambridge Cognitive Examination-Revised; L = Linear Model; MMSE = Mini-Mental State Exam ; OCSP = Oxford Community Stroke project classification; PiB = Pittsburgh Compound-B; SF = Step-Function models; VICCCS = Vascular Impairment of Cognition Classification Consensus Study; PACS = Partial Anterior Circulation Stroke; TACS = Total Anterior Circulation Stroke Syndrome; POCS = posterior circulation stroke

For Review Only

Introduction

In the UK, over 100,000 cases of stroke occur each year (117,600 in 2015) and this is expected to increase by 60% by 2035 (*n=186,900*).[1] Stroke remains a leading cause of death, long-term disability and cognitive impairment.[2,3] There is an established link between incident stroke and cognitive decline, namely the development of vascular dementia, with around 10% of individuals developing dementia following a first-ever stroke, and 30% after recurrent stroke.[2]

Stroke may bring forward a dementia diagnosis by 10 years.[4,5] Levine et al (2015) demonstrated that an acute decline in cognition at the time of stroke is followed by a persistent linear decline in the following years.[6] However, the mechanisms and the direct effects of stroke on global cognition, as well as on executive function leading up to dementia remain poorly understood,[4] as is the relationship between brain injury caused by stroke, brain reserve, the ability for the brain to withstand injury, and onset of dementia.[5] Risk factors have been proposed for post-stroke dementia, outside of markers for stroke complications, include age, female sex, low education, race, diabetes and atrial fibrillation which are also known risk factors for Alzheimer's dementia or pre-stroke dementia.[7,8] Also, linear trajectories for post-stroke cognitive decline described previously are at odds with the descriptions of cognitive decline for vascular dementia.[6,8] A linear trajectory describes a progressive decline that is more characteristic of Alzheimer's dementia, while vascular dementia is ascribed a fluctuating,[9] or a stepwise cognitive decline towards dementia.[10,11] This latter trajectory is consistent with findings that multiple and recurrent strokes are predictive of dementia.[8]

There remain significant gaps in our understanding of cognitive decline following a stroke, and how it may lead, or describe progression towards dementia.[8] We explored cognitive function trajectories in our longitudinal prospective study of elderly stroke survivors.[3] In the Newcastle Cognitive Function after Stroke (CogFAST) cohort, we previously showed that more than 75%

For Review Only

of stroke survivors develop vascular dementia meeting criteria for severe vascular cognitive impairment per the VICCCS consortium criteria.[12] Here, we investigated: 1) trajectory of cognitive decline in following stroke, in individuals that develop dementia against those who do not, 2) characteristics of cognitive change in post-stroke survivors who develop dementia, and 3) and risk factors precipitating onset of dementia.[3]

Materials and methods

The CogFAST cohort is a secondary-care-based longitudinal study of older people (aged 75 year and older) who were diagnosed with stroke in hospital and established by neuroimaging.[3] Stroke was defined according to the World Health Organization definition and classified according to the Oxford Community Stroke project classification.[3] We focussed on 355 individuals without dementia at baseline (3 months post-stroke) that were followed until diagnosed with dementia or loss to follow-up. For this study, we included up to 12 years of follow-up, the last year of follow-up with a diagnosis of dementia. Participants were separated into to two groups: 1) a “dementia” group includes all individuals that develop dementia at any time during the 12 year follow-up, 2) a comparison group, named “no dementia” that did not develop dementia during follow-up.

Participants were free from dementia at baseline and from disabilities precluding computer-assisted cognitive testing (e.g. aphasia, hemiparesis affecting the hand used for writing).[13] Participants received annual clinical and neuro-psychologic assessments. CAMCOG-R is a standardized test for global cognitive performance (maximum score of 107), subdivided into 10 domains for memory (27 points), orientation (10 points), language comprehension (9 points), language expression (21 points), attention (7 points), praxis (12 points), calculation (2 points), abstract thinking (8 points) perception (11 points).[3,14] A separate executive function domain was scored out of 28 points. However, this new item did not contribute to the total

For Review Only

CAMCOG-R score. MMSE provides a quantitative estimate of severity of cognitive impairment" (maximum of 30 points), based on questions covering 7 domains: Orientation to time (5 points); Orientation to place (5 points); Registration of three words (3 points); Attention and Calculation (5 points); Recall of three words (3 points); Language (8 points) and Visual Construction (1 point). The items for the MMSE in this study were embedded in the CAMCOG R assessment. IQ at baseline was assessed using the National Adult Reading Test.[15]

Statistical analysis

Trajectories of cognitive change

Trajectories of cognitive function, as measured by the CAMCOG and MMSE during the follow-up period, were modelled as a function of the years from baseline until an individual's end of follow-up. We used random linear mixed-effects models, fitted via maximum likelihood with random effects specified at individual level. Analysis also included adjustments for age (<80 and ≥80 years) and sex.[16] Three linear models (L) were produced: L1) full cohort for full duration of the follow-up; L2) full cohort with an interaction term for diagnosis of dementia at end of follow-up; and L3) full cohort with an interaction term for dementia and censoring of the last years of follow-up.

For Review Only

For the dementia group, we produced analysis stratifying participants into 4 groups by length of follow-up, which includes individuals with up to 1 to 3 years, up to 4 to 6 years (Y), up to 7 to 9 years and up to 10 to 12 years of follow-up time. These were name 0-3Y, 4-6Y, 7-8Y and 10-12Y, respectively. We used random linear mixed-effects models, with follow-up years as indicator variables to account for the nonlinear form of trajectories (hereafter referred to as the step function model - SF). This implementation compares each year of follow-up against a baseline value, allowing for identifying possible inflections points that are concealed by linear

models. Two sets of step-function models were produced: SF1) complete follow-up and SF2) censoring the last 3 years of follow-up.

Results from mixed effect models are displayed as profile plots, produced using population marginal means or predicted means.[16] These are estimated from the fitted model, and preferred to observed means, which do not account for the underlying model of the data.[17,18] Profile plots are useful for comparing marginal means in the model, where a line plot in which each point indicates the estimated marginal mean of a dependent variable (adjusted for covariates) at one level of a factor.

For Review Only

Declines in CAMCOG during follow-up

For people with dementia, we analysed where within in the follow-up time large reductions in CAMCOG score from one year of follow to the next were more common. We estimated the proportion of individuals that had a decline in CAMCOG score of a) at least five points, and b) at least 10 point at 4 stages of follow-up starting in the 3 year before diagnosis, 4 to 6, 7 to 8 and 10 to 12 years before diagnosis. Analyses were performed separately for each follow-up category for the 0-3Y, 4-6Y, 7-8Y and 10-12Y groups.

Risk factors of step cognitive decline

We analysed risk factors for early onset of cognitive decline, defined as the group of participants that developed dementia within 3 years after stroke, compared to those that developed after 3 or more years. risk factors include: OCSP classification, cognitive impairment no dementia (CIND), equivalent to mild vascular cognitive impairment,[12] previous stroke, previous disabling stroke, apolipoprotein E (*APOE*) ε4, hypertension, myocardial infarction, ischaemic heart disease, type 2 diabetes, atrial fibrillation, hypercholesterolemia, smoking history and number of risk factors (SD). We used logistic regression models adjusted for age and sex.

Analyses were performed using STATA version 15, 2017. Stata Statistical Software: Release 15. StataCorp LLC. For all analysis, statistical significance was set at p-value <0.05.

Data availability

The data that support the findings of this study are available on request from the corresponding or senior authors. The data are not publicly available due to privacy or ethical restrictions.

Results

Of the *n=355* participants enrolled in the CogFAST study between 1999 and 2003, *n=91 (25.6%)* individuals developed dementia during follow-up (Table 1). The two groups were comparable in age *(p=0.66)* and sex *(p=0.05)*, stroke types *(p=0.83)*, with predominantly ischaemic infarction in both groups, and IQ *(p=0.12)*. The dementia group had lower CAMCOG-R scores at baseline *(p <0.01)* although not diagnostic for dementia and at the end of follow-up *(p<0.01)*. The dementia group was followed for a total of *310 years (Mean=3.4, SD = 2.6)* and the non-dementia group for a total of *900 years (Mean=3.4, SD = 3.3).*

Change in cognition after stroke: linear model

CAMCOG changed an average of *0.68 (CI95%=-0.81:-0.54, p<0.001)* points of year of follow-up in the L1 models (Figure 1a and Supplementary Table 1). The L2 model, with interaction terms for dementia identified a steeper yearly decline in CAMCOG scores in the dementia group compared to the no dementia group *(coef.=-1.91, CI95%=-2.23:-1.59, p<0.01)* with the dementia groups starting from lower score at baseline *(coef.=-2.46, CI95%=-3.11:-1.81, p<0.01*- Figure 1b). The L1 model applied to MMSE found a similar trend with combined groups showing a yearly decline of *-0.32 (CI95%=-0.37:-0.26, p<0.01* - Figure 2a and Table S2), while the L2 model with interaction term, also found the dementia group had a steeper

For Review Only

yearly decline *(coef.=-0.46, CI95%=-0.58:-0.34, p<0.01)*, as well as a lower score at baseline (*coef.=-2.46, CI95%=-3.11:-1.81, p.<0.01* – Figure 2b and Supplementary Table 2).

Change in cognition in the dementia group

The SF1 model describes CAMCOG trajectories, stratified by length of follow-up (Figure 3a). All four groups showed a decline in CAMCOG over the full follow-up period (*1-3Y coef=-15.24, CI95%=-19.78:-10.70, p<0.01; 4-6Y coef=-17.43 CI95%=-22.29:-12.57, p<0.01, 7-9Y coef.=-20.21, CI95%=-25.54:-14.88, p<0.01; 10-12Y coef.=-33.49, CI95%=-41.91:-25.07, p<0.01* - Table S3). The 1-3 year group demonstrated stepwise decline in CAMCOG score over the 3 years of follow-up (Figure 3a). However, groups with longer follow-ups showed a period of cognitive stability where CAMCOG was not statistically different from baseline, lasting 3 years for the 4-6Y *(coef.=0.28, CI95%=-3.28:3.8, p=0.88)*; 4 years for the 7-9Y *(coef.=-3.00, CI95%=-6.45:0.45, p=0.09)* and 6 years for the 10-12Y *(coef.=-6.50, CI95%=-13.27:0.27, p=0.06*). This was followed by a steep decline in the last 3 years of follow-up (Figure 3a and Supplementary Table 5). Application of the SF1 model to MMSE produced similar results, with a period of cognitive stability of 3 years 4-6Y *(coef.=-0.19, CI95%=-1.44:1.06, p=0.764)*, 5 years for the 7-9Y *(coef.=-1.29, CI95%=3.23:0.66, p=0.196)*, 8 years for the 10-12Y *(coef.-1.75, CI95%=-4.30:0.80, p=0.178)*, with cognitive decline occurring in the last 3 years of follow-up (Figure 4a and Supplementary Table 7).

Decline was also observed in all individual CAMCOG components in the last three years of follow-up (Table 2 & Supplementary Figure 1). The greatest yearly declines were observed for orientation with a *-7.06% (CI95%=-8.62:-5.51, p<0.001)*, *-6.04%* for total memory *(CI95%=-7.14:-4.95, p<0.001)*, *-4.52%* for attention *(CI95%=-6.37:-2.67, p<0.001*) and *-4.09%* for praxis *(CI95%=-5.49:-2.69, p<0.001)*.

For Review Only

Follow-up with last 3 years censored

The SF2 model, with censoring of the least three years of follow-up, showed trajectories of cognitive change remain mostly stable throughout the follow-up period with little decline at the end of follow-up *(4-6Y: coef.=2.77, CI95%=1.30:6.85, p=0.18; 7-9Y: coef.=-8.43 CI95%=-12.28:-4.58, p<0.01; 10-12Y: Coef=-9.56, CI95%=-16.63:-2.49, p<0.01* - Supplementary Table 6 and Figure 3b). The SF2 model applied to MMSE showed similar results *(4-6Y: coef.=1.04 CI95%=-0.13-2.21, p.<0.083; 7-9Y: coef.=-1.78, CI95%=-4.26:0.70, p<0.160; 10-12Y coef.=-3.00, CI95%=-5.62:-0.37, p<0.025* - Figure 4b and Supplementary Table 8). The 1-3Y group was excluded from the SF2 model as no observations were available after censoring.

The L3 models, with interaction term and with censoring of the last three years of follow-up for the dementia group, showed trajectories of cognitive change were only marginally different between the dementia and no dementia group for CAMCOG *(coef.=-0.49 CI -0.97:0.00, p=0.048*- Supplementary Table 1*)* and not statistically different for MMSE *(coef.=0.03 CI -0.16:0.21, p=0.788* – Supplementary Table 2*)*.

For Review Only

Precipitating factors for onset of dementia in first 3 years

Univariate models produced to identify risk of developing dementia within 3 years of follow-up identified history of recurrent stroke was associated with increased risk of developing dementia within 3 years after the event *(OR=3.99 CI95%=1.30:12.25, p=0.016)*. All individuals with a history of previously disabling stroke developed dementia within 3 years, indicating an infinite OR (Table 3). Likelihood of diagnosis of dementia increased with increasing number of risk factors *(OR=2.02 CI95%=1.26:3.25, p=0.004)*, although a stratified analysis on number of risk factors was not significant (Table 3). CIND at baseline was also associated with increased risk of dementia within 3 years *(OR=3.50 CI95%=1.29:9.49,*

p=0.014). No other risk factors were statistically significant although few trends were noteworthy including type 2 diabetes *(OR=4.50 CI95%=0.53:38.36, p=0.169)*, hypercholesterolemia *(OR=3.44 CI95%=0.38:31.56, p=0.274)* and hypertension *(OR 2.31 CI95%=0.92:5.81, p<0.074)* (Table 3).

Lastly, compared to lacunar stroke, partial anterior circulation stroke (PACS) show a non-significant association with dementia within 3 years *(OR=1.15 CI95%=0.41:3.25, p=0.789)* and total anterior circulation stroke syndrome (TACS) showed a stronger relationship still *(OR=3.18 CI95%=0.33:30.55, p=0.317)*, while those with posterior circulation stroke (POCS) seemed to indicate reduced risk of dementia within 3 years *(OR 0.48 CI 0.11:2.15, p=0.339)*. The multivariate analysis attenuated most odds ratios with none of the candidate risk factors achieving significance. *APOE* ε4 was not associated with a diagnosis of dementia within 3 years (Table 3).

Discussion

Our unique, large study in older (≥75 years age) stroke survivors in the North East of England characterised the relationship between trajectories of cognitive function and post-stroke dementia during up to 12 years of follow-up in a hospital-based cohort of survivors of first or recurrent stroke. It showed that decline in global cognition following stroke follows a pattern of cognitive decline that can be more precisely described as fluctuating,[9] or stepwise.[10,11] Such a pattern is traditionally associated with VaD caused by multiple infarcts.

In the dementia group, the SF1 model showed a steep linear decline in global cognition for those developing dementia within the first 3 years of follow-up. However, individuals with longer follow-up had a period of cognitive stability immediately after stroke which could last up to 8 years, with steep declines starting only in the 3 years before a dementia diagnosis. After

For Review Only

excluding the last 3 years of follow-up in the dementia group (SF2 model), these individuals showed similar, mostly flat trajectories of cognitive function regardless of follow-up time. Most importantly, in the linear models where the dementia groups showed a steeper decline global cognition compared to the no-dementia for CAMCOG-R and MMSE (L2 model), after excluding the last 3 years of follow-up, the cognitive trajectory from the dementia group was not dissimilar, or only modestly dissimilar from that of the no-dementia group. These findings contradict previous characterisations of progressive but persistent cognitive decline.[6] They also suggest these individuals may not yet have been on a path towards dementia, and thus while stroke may predispose for dementia, cognitive decline may only occur after a subsequent precipitating event, i.e. a further assault or injury to the brain.

Data on incident stroke and other potential causes for brain injury were not collected during follow-up and therefore we cannot identify whether subsequent milder or covert events, which may have not required hospitalisation precipitated cognitive decline. Nonetheless, a risk factor analysis for developing dementia within 3 years of stroke (versus more than 3 years) found a history of previous stroke at baseline was a precipitating factor for dementia, which is consistent with previous findings.[3] Severity of stroke was also a precipitating factor. For example, all individuals with a record of disabling stroke developed dementia within 3 years, while stroke types, based OCSP classification displayed a dose-response trend where strokes causing the greatest damage to the frontal-temporal area were more likely to result in dementia within 3 years.[8] Remarkably, the presence of the *APOE* ε4 was not associated with the onset of dementia in 3 years.[19] This appears consistent that post-stroke survivors develop VaD and lack Alzheimer type of pathology or amyloid burden as determined by PiB binding in more than 70% of post-stroke survivors.[3,20]

Our findings are in accord with the concept of brain reserve, in this case, the brain's ability to sustain a certain level of stroke injury before clinical or cognitive deficit emerges.[5,21] Literature

For Review Only

on cognitive reserve highlights the severity of the injury, and the degree of cumulative lesion burden as factors in cognitive outcomes.[5] Our findings show those diagnosed with dementia within 3 years had evidence of previous brain injury, i.e. previous stroke, or suffered from an overt stroke, causing depletion of brain reserve and cognitive decline. In contrast, those diagnosed with dementia after more than 3 years were more likely to have milder forms of stroke. This concept and our finding suggest that individuals with previous but not debilitating stroke have a predisposition to cognitive decline, where the brain can function normally but in a state of heightened susceptibility to additional injury. In cases where additional injury occurs, it can precipitate relatively rapid cognitive decline towards dementia. Clinically, this has implications for both the follow up of people who have had a stroke and for history taking in memory clinic settings. Primary care physicians should be aware that those who appear to be stable after a stroke may subsequently go on to develop further episodes of stroke or brain injury and they need to enquire about cognitive step-down at routine annual reviews of these patients. In the memory clinic setting, it is useful to enquire about the pattern of cognitive decline after stroke and be aware that further brain events can cause a stepdown in cognition which point to a vascular cause of dementia. Clinicians should ask about further stroke symptoms even if these are transient or covert events possibly uncovered by re-imaging.[22] Furthermore, controlling vascular risk factors in these patients should be vigorously pursued.[3]

Examination of cognitive domains showed that trajectories were steeper in orientation, memory, attention and praxis. It is not surprising that memory was particularly affected as we used a definition of dementia which includes change in memory as a criterion. However, the changes in attention and praxis may be domains which the clinician could particularly look out for during follow up. It is not unlikely that deficits in these cognitive domains reflect the progressive disruption of the fronto-subcortical circuits also suggested by aberrations in the default mode network.[23] Both orientation or spatial navigation neglect and memory may be

For Review Only

associated with the disconnection with the temporal lobe, in particular, the hippocampal formation.[24]

Strengths and limitations.

Our distinctive study provides a longitudinal analysis of change in global cognition following stroke and followed individuals. It includes a sample of *n=355* participants that were followed for up to 12 years, the longest follow-up available in a cohort study focusing on post-stroke dementia. Cognitive function was measured with two standardised and peer-reviewed methods the CAMCOG-R and the MMSE score. Analysis of trajectories of cognitive decline produced in L and SF models were replicated across both measures of cognitive decline.

There are few limitations to this study. We found only 91 of the 355 participants were diagnosed with dementia. This impacts analysis focusing on a limited number of survival groups. Ideally, in SF1 and SF2 analysis should have included 12 groups, stratifying by each year follow-up duration. However, this was not possible due to sample size limitations. The compromise solution was stratifying participants into 4 groups that still allowed detection of differences by length of follow-up time. This also impacted the available statistical power to identify risk factors for diagnosis of dementia within 3 years. Survivorship bias is a possible limitation, as some participants in the non-dementia group may have been lost to follow-up before developing dementia. This is unlikely to have affected the analysis of trajectories where the non-dementia group sustained cognitive function throughout follow-up, or the analysis of risk factors which we limited to the dementia group. Lastly it was not possible within the resources of the study to establish, with accuracy, whether further strokes and other events able to cause brain damage, had occurred during follow-up.

For Review Only

Conclusions

This unique large study in older (≥75 years age) stroke survivors produced evidence that older people who experience stroke can have a period of stable cognitive function for a number of years after stroke. However, the notable finding is that post-stroke survivors undergo a remarkable decline approximately 3 years before the dementia threshold. Collectively, this can then be followed by a stepwise decline which should alert the clinician to a possibility of impending VaD.

Acknowledgements

Acknowledgements

We are grateful to the patients, families, and clinical house staff for their cooperation in the investigation of this study. We thank Michelle Widdrington, Carein Todd, Jean Scott, Deborah Lett, Anne Nicholson and Sally Stephens for assistance in managing and screening the cohort set up as the cognitive function after stroke (CogFAST) study.

Funding

Our work was supported by grants from the UK Medical Research Council (MRC, G0500247 and G0700718) for the Vascular Factors in Neurodegeneration and Dementia study and theNewcastle Centre for Brain Ageing and Vitality (BBSRC, EPSRC, ESRC and MRC, LLHW G0700718).

LA is supported by the National Institute for Health Research Applied Research Collaboration South West Peninsula. The views expressed in this publication are those of the authors and not necessarily those of the National Institute for Health Research or the Department of Health and Social Care.

JM is funded by the National Institute for Health Research (NIHR), (NIHR Development and Skills Enhancement Fellowship, NIHR301445). This publication presents independent research funded by the National Institute for Health Research (NIHR). The views expressed

For Review Only

are those of the author(s) and not necessarily those of the NHS, the NIHR or the Department of Health and Social Care.

Competing interests

The authors report no competing interests.

Supplementary material

Supplementary material is available at *Brain* online.

For Review Only

References

1. King D, Wittenberg R, Patel A, Quayyum Z, Berdunov V, Knapp M. The future incidence, prevalence and costs of stroke in the UK. *Age Ageing*. 2020;49(2):277-282. doi:10.1093/ageing/afz163
2. Kalaria RN, Akinyemi R, Ihara M. Stroke injury, cognitive impairment and vascular dementia. *Biochim Biophys Acta - Mol Basis Dis*. 2016;1862(5):915-925. doi:10.1016/j.bbadis.2016.01.015
3. Allan LM, Rowan EN, Firbank MJ, et al. Long term incidence of dementia, predictors of mortality and pathological diagnosis in older stroke survivors. *Brain*. 2011;134(12):3713-3724. doi:10.1093/brain/awr273
4. Mijajlović MD, Pavlović A, Brainin M, et al. Post-stroke dementia - a comprehensive review. *BMC Med*. 2017;15(1):1-12. doi:10.1186/s12916-017-0779-7
5. Bigler ED, Stern Y. *Traumatic Brain Injury and Reserve*. Vol 128. 1st ed. Elsevier Ltd.; 2015. doi:10.1016/B978-0-444-63521-1.00043-1
6. Levine DA, Galecki AT, Langa KM, et al. Trajectory of cognitive decline after incident stroke. *JAMA - J Am Med Assoc*. 2015;314(1):41-51. doi:10.1001/jama.2015.6968
7. De Ronchi D, Palmer K, Pioggiosi P, et al. The combined effect of age, education, and stroke on dementia and cognitive impairment no dementia in the elderly. *Dement Geriatr Cogn Disord*. 2007;24(4):266-273. doi:10.1159/000107102
8. Pendlebury ST, Rothwell PM. Prevalence, incidence, and factors associated with pre-stroke and post-stroke dementia: a systematic review and meta-analysis. *Lancet Neurol*. 2009;8(11):1006-1018. doi:10.1016/S1474-4422(09)70236-4
9. Del Ser T, Barba R, Morin MM, et al. Evolution of cognitive impairment after stroke and risk factors for delayed progression. *Stroke*. 2005;36(12):2670-2675. doi:10.1161/01.STR.0000189626.71033.35
10. Chui HC, Victoroff JI, Margolin M, Jagust W, Shankle R, Katzman R. Criteria for the diagnosis of ischemic vascular dementia proposed by the state of California Alzheimer's disease diagnostic and treatment centers. *Neurology*. 1992;42(3):473-480. doi:10.1212/wnl.42.3.473
11. Román GC, Tatemichi TK, Erkinjuntti T, et al. Vascular dementia: Diagnostic criteria for research studies: Report of the ninds-airen international workshop*. *Neurology*. 1993;43(2):250-260. doi:10.1212/wnl.43.2.250
12. Skrobot OA, Black SE, Chen C, et al. Progress toward standardized diagnosis of

For Review Only

vascular cognitive impairment: Guidelines from the Vascular Impairment of Cognition Classification Consensus Study. *Alzheimer's Dement*. 2018;14(3):280-292. doi:10.1016/j.jalz.2017.09.007

13. American Psychiatric Association. *Diagnostic and Statistical Manual of Mental Disorders*. Fifth.; 2013.
14. Roth M, Tym E, Mountjoy CQ, et al. CAMDEX. A standardised instrument for the diagnosis of mental disorder in the elderly with special reference to the early detection of dementia. *Br J Psychiatry*. 1986;149(DEC.):698-709. doi:10.1192/bjp.149.6.698
15. Bright P, Jaldow E, Kopelman MD. The National Adult Reading Test as a measure of premorbid intelligence: A comparison with estimates derived from demographic variables. *J Int Neuropsychol Soc*. 2002;8(6):847-854. doi:10.1017/S1355617702860131
16. Alley DE, Metter EJ, Griswold ME, et al. Changes in weight at the end of life: Characterizing weight loss by time to death in a cohort study of older men. *Am J Epidemiol*. 2010;172(5):558-565. doi:10.1093/aje/kwq168
17. Williams R. Using the margins command to estimate and interpret adjusted predictions and marginal effects. *Stata J*. 2012;12(2):308-331. doi:10.1177/1536867x1201200209
18. Royston P. Marginscontplot: Plotting the marginal effects of continuous predictors. *Stata J*. 2013;13(3):510-527. doi:10.1177/1536867x1301300305
19. Ballard CG, Morris CM, Rao H, et al. APOE ε4 and cognitive decline in older stroke patients with early cognitive impairment. *Neurology*. 2004;63(8):1399-1402. doi:10.1212/01.WNL.0000141851.93193.17
20. Mok V, Leung EYL, Chu W, et al. Pittsburgh compound B binding in poststroke dementia. *J Neurol Sci*. 2010;290(1-2):135-137. doi:10.1016/j.jns.2009.12.014
21. Stern Y, Barulli D. Cognitive reserve. In: *Handbook of Clinical Neurology*. Vol 167. ; 2019:181-190. doi:10.1016/B978-0-12-804766-8.00011-X
22. Debette S, Schilling S, Duperron MG, Larsson SC, Markus HS. Clinical Significance of Magnetic Resonance Imaging Markers of Vascular Brain Injury: A Systematic Review and Meta-analysis. *JAMA Neurol*. 2019;76(1):81-94. doi:10.1001/jamaneurol.2018.3122
23. Jobson DD, Hase Y, Clarkson AN, Kalaria RN. The role of the medial prefrontal cortex in cognition, ageing and dementia. *Brain Commun*. 2021;3(3):fcab125. doi:10.1093/braincomms/fcab125
24. Gemmell E, Bosomworth H, Allan L, et al. Hippocampal neuronal atrophy and cognitive

For Review Only

function in delayed poststroke and aging-related dementias. *Stroke*. 2012;43(3):808-814. doi:10.1161/STROKEAHA.111.636498

For Review Only

Figure legends

Figure 1: Trajectory of CAMCOG score per year of follow-up a) complete cohort, b) by dementia status at end of follow-up c) same as b but with censoring of last 3 years of follow-up

Figure 2: Trajectory of MMSE score per year of follow-up a) complete cohort, b) by dementia status at end of follow-up c) same as b but with censoring of last 3 years of follow-up.

Figure 3: CAMCOG score per year of follow-up in the incident dementia group a) stratified by length of follow-up, in 3 year segments b) same as a) but with censoring of last 3 years of follow-up.

Figure 4: MMSE score per year of follow-up in the incident dementia group a) stratified by length of follow-up, in 3 year segments b) same as a but with censoring of last 3 years of follow-up.

For Review Only

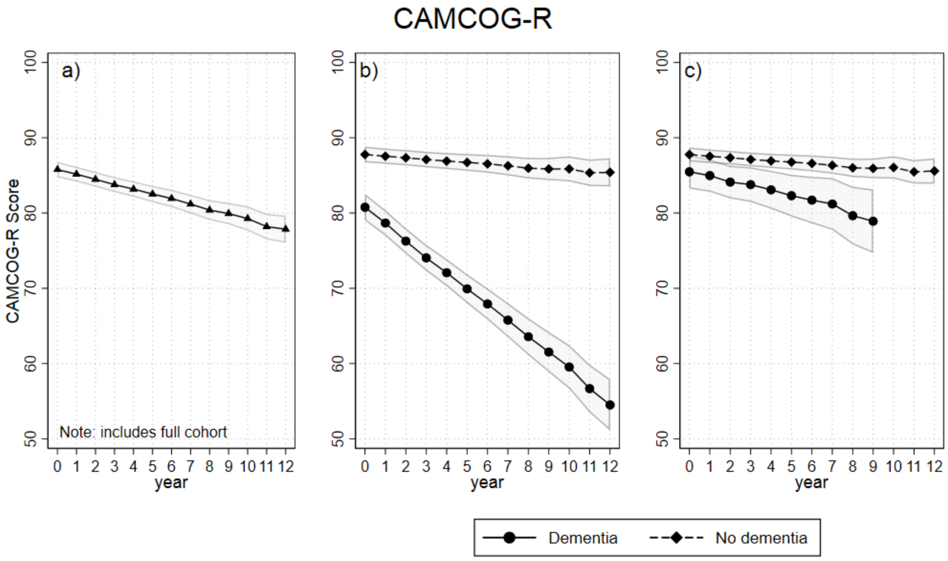

Figure 1: Trajectory of CAMCOG score per year of follow-up a) complete cohort, b) by dementia status at end of follow-up c) same as b but with censoring of last 3 years of follow-up.

368x216mm (72 x 72 DPI)

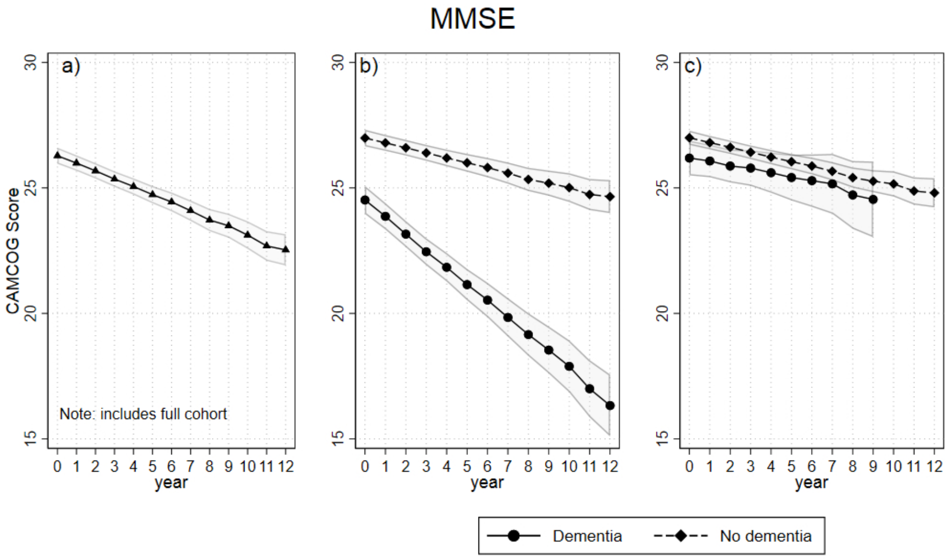

Figure 2: Trajectory of MMSE score per year of follow-up a) complete cohort, b) by dementia status at end of follow-up c) same as b but with censoring of last 3 years of follow-up.

368x216mm (72 x 72 DPI)

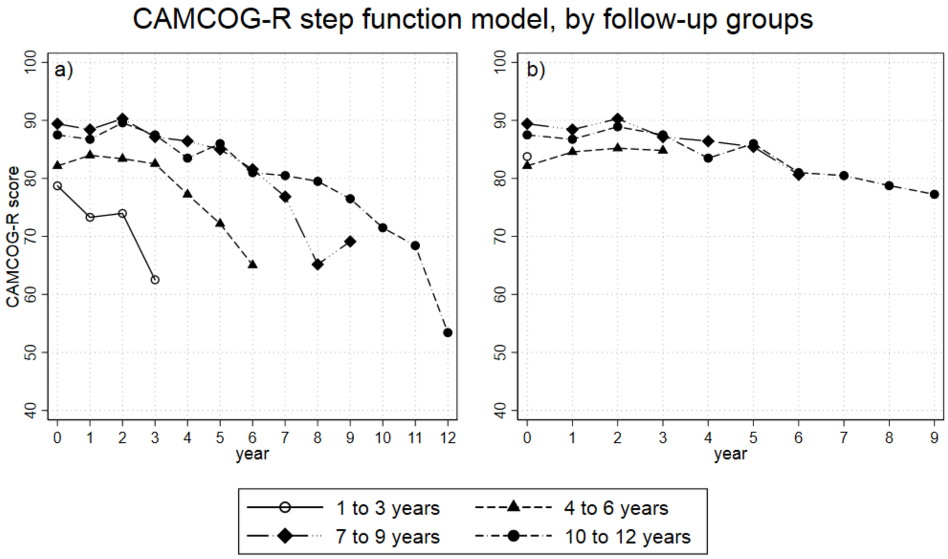

Figure 3: CAMCOG score per year of follow-up in the incident dementia group a) stratified by length of follow-up, in 3 year segments b) same as a) but with censoring of last 3 years of follow-up.

368x216mm (72 x 72 DPI)

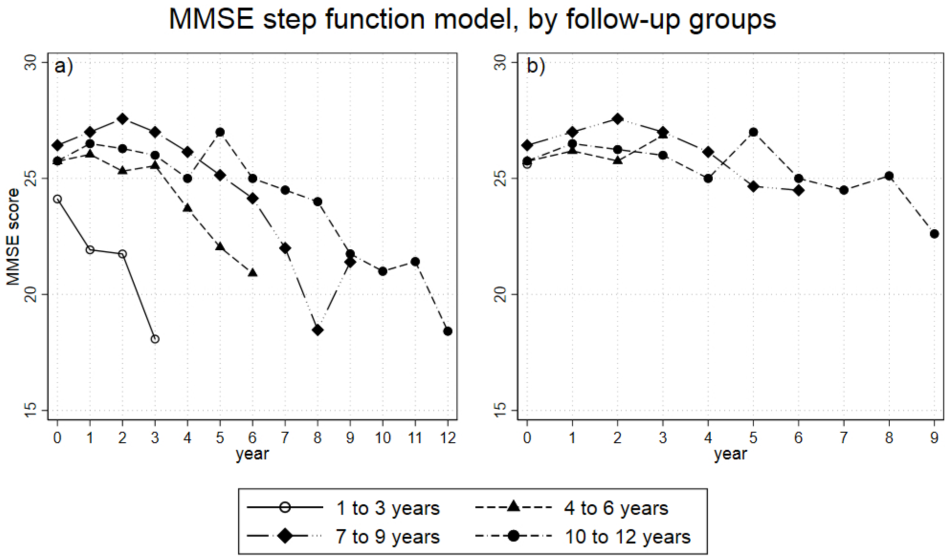

Figure 4: MMSE score per year of follow-up in the incident dementia group a) stratified by length of follow-up, in 3 year segments b) same as a but with censoring of last 3 years of follow-up.

368x216mm (72 x 72 DPI)

Tables

Table 1: Demographic characteristics

Variable	Dementia	No dementia	p value
Number	91	264	
Female (%)	52 (57.1)	119 (45.1)	0.05
Age at baseline	80.3(4.5)	80.1(4)	0.66
Final diagnosis	-	-	
Ischaemic infarction	83 (91.2)	228 (86.4)	0.828
Haemorrhagic infarction	2 (2.2)	4 (1.5)	-
Intracerebral haemorrhage	2 (2.2)	9 (3.4)	-
TIA	1 (1.1)	7 (2.7)	-
Multiple	0 (0)	1 (0.4)	-
Not known	0 (0)	2 (0.8)	-
Missing	3 (3.3)	13 (4.9)	-
Cognitive measures			
CAMCOG-R (baseline)	80.6 (9.3)	86.6 (8.3)	<0.01
CAMCOG-R (end of follow-up)	59.2 (16.8)	86.7 (9.8)	<0.01
Change in CAMCOG-R	21.6 (17.7)	1.2 (9.6)	<0.01
MMSE (baseline)	24.7 (3.1)	26.6 (2.5)	<0.01
MMSE (end of follow-up)	20.1 (3.9)	25.8 (3.5)	<0.01
Change in MMSE	-4.6 (4.3)	-0.8 (3.1)	<0.01
Full scale IQ	105.9 (12.3)	108.2 (10.8)	0.12
Verbal IQ	104.6 (11.3)	106.7 (10)	0.12
Follow-up in years	-	-	-
Baseline only	0 (0.0)	58 (22.0)	<0.01
1 to 3	61 (67.0)	105 (39.8)	-
4 to 6	19 (20.9)	47 (17.8)	-
7 to 9	7 (7.7)	41 (15.5)	-
10 to 12	4 (4.4)	13 (4.9)	-
OCSP classification	-	-	-
LACS	29(31.9)	87(33.0)	0.78
PACS	38(41.8)	107(40.5)	-
TACS	7(7.7)	12(4.5)	-
POCS	10(11.0)	39(14.8)	-
Not classified	7(7.7)	19(7.2)	
CIND (CAMCOG-R <80)	31(50.8)	7(23.3)	-

For Review Only

Table 2: Change in CAMCOG component in the last 3 year of follow in the dementia group.

		Total Score				Percentage change			
	Max	coef.	LL	UL	p-val.	%	LL	UL	p-val.
CAMCOG-R	107	-4.6	-5.5	-3.8	0.00	-4.4	-5.1	-3.6	0.00
Orientation	10	-0.7	-0.9	-0.6	0.00	-7.1	-8.6	-5.5	0.00
Memory total	27	-1.6	-1.9	-1.3	0.00	-6.0	-7.1	-5.0	0.00
Language total	30	-0.7	-0.9	-0.5	0.00	-2.3	-3.0	-1.5	0.00
Attention	9	-0.4	-0.6	-0.2	0.00	-4.5	-6.4	-2.7	0.00
Praxis	12	-0.5	-0.7	-0.3	0.00	-4.1	-5.5	-2.7	0.00
Perception	10	-0.3	-0.4	-0.1	0.00	-2.8	-4.2	-1.4	0.00
Abstract thinking	8	-0.2	-0.3	0.0	0.08	-2.0	-4.2	0.3	0.08
Total executive*	28	-0.7	-1.1	-0.4	0.00	-2.6	-3.8	-1.5	0.00

* Does not contrite to total CAMCOG-R score.

Table 3: Univariate and multivariate predictors of death

	Dementia in		Univariate model				Multivariate model			
	≤ 3 years	> 3 years	OR	LL	UL	p-val	OR	LL	UL	p-val
Number	-	-	-	-	-	-	-	-	-	-
OCSP classification	-	-	-	-	-	-	-	-	-	-
LACS	19(31.1)	10(33.3)	ref	-	-	-	-	-	-	-
PACS	26(42.6)	12(40.0)	1.15	0.41	3.25	0.789	0.77	0.23	2.57	0.665
TACS	6(9.8)	1(3.3)	3.18	0.33	30.55	0.317	4.61	0.34	62.01	0.249
POCS	5(8.2)	5(16.7)	0.48	0.11	2.15	0.339	0.26	0.04	1.78	0.168
Number of risk factors (SD)*	-	-	-	-	-	-	-	-	-	-
0	3(10.0)	6(9.8)	ref	-	-	-	-	-	-	-
1	15(50.0)	12(19.7)	0.38	0.08	1.90	0.240	-	-	-	-
2	9(30.0)	20(32.8)	1.07	0.22	5.33	0.933	-	-	-	-
3 or more	3(10.0)	23(37.7)	3.80	0.60	23.88	0.155	-	-	-	-
Count of risk factors	-	-	2.02	1.26	3.25	0.004				
Previous stroke	25(41.0)	5(16.7)	3.99	1.30	12.25	0.016	3.74	0.91	15.39	0.068
Previous disabling stroke*	15(24.6)	0(0.0)	-	-	-	-	-	-	-	-
CIND	31(50.8)	7(23.3)	3.50	1.29	9.49	0.014	3.00	0.86	10.40	0.084
APOE ε4	13(21.3)	9(30.0)	0.60	0.22	1.65	0.320	1.15	0.30	4.39	0.843
Hypertension	39(63.9)	14(46.7)	2.31	0.92	5.81	0.074	3.07	0.91	10.39	0.072
Myocardial Infarction	14(23.0)	6(20.0)	1.32	0.43	4.00	0.628	1.14	0.28	4.63	0.855
Ischaemic Heart Disease	25(41.0)	11(36.7)	1.29	0.52	3.20	0.583	1.14	0.32	4.08	0.846
Type 2 Diabetes	8(13.1)	1(3.3)	4.50	0.53	38.36	0.169	6.14	0.36	105.23	0.211
Atrial Fibrillation	11(18.0)	4(13.3)	1.50	0.43	5.18	0.525	1.34	0.22	8.03	0.746
Hypercholesterolemia	6(9.8)	1(3.3)	3.44	0.38	31.56	0.274	1.00			
Smoking history	38(62.3)	18(60.0)	1.08	0.41	2.85	0.878	1.04	0.26	4.08	0.958
Age	-	-	0.98	0.89	1.08	0.66	0.95	0.83	1.09	0.499
Sex	-	-	0.97	0.40	2.35	0.95	1.36	0.39	4.78	0.631

* Excluded from multivariate model due to possible over-adjustment.

For Review Only

Supplemental materials

Tables

Supplementary Table 1: Linear mixed effect model for CAMCOG-R per year of follow-up (left) with an interaction term on incident dementia (left)

	a) Simple model		b) interaction on dementia		c) 3 years censored	
	coef.	CI 95%	coef.	CI 95%	coef.	CI 95%
Year	-0.68*	-0.82:-0.54	-0.25*	-0.38:-0.12	-0.25*	-0.38:-0.12
Dementia	-	-:-	-7.1*	-9.16:-5.05	-2.11	-4.52:0.31
Dementia + Year	-	-:-	-1.91*	-2.23:-1.59	-0.49*	-0.97:0.00
Age	-2.07*	-4.03:-0.11	-2.43*	-4.16:-0.7	-3.05*	-4.70:-1.39
Gender	1.61	-0.36:3.57	0.38	-1.36:2.13	0.23	-1.43:1.90
Constant	84.35*	80.97:87.72	88.42*	85.31:91.52	88.96*	86.02:91.90

* P-values <0.05

Supplementary Table 2: Linear mixed effect model for MMSE per year of follow-up (left) with an interaction term on incident dementia (left)

	a) Simple model		b) interaction on dementia		c) 3 years censored	
	coef.	CI 95%	coef.	CI 95%	coef.	CI 95%
Year	-0.32*	-0.37:-0.26	-0.21*	-0.26:-0.15	-0.20*	-0.25:-0.15
Dementia			-2.46*	-3.11:-1.81	-0.74	-1.48:0.01
Dementia + Year			-0.46*	-0.58:-0.34	0.03	-0.16:0.21
Age	-0.44	-1.05:0.17	-0.57*	-1.10:-0.03	-0.83*	-1.33:-0.34
Gender	0.73*	0.12:1.34	0.33	-0.21:0.87	0.22	-0.27:0.72
Constant	25.40*	24.34:26.45	26.76*	25.80:27.73	27.08*	26.20:27.95

* P-values <0.05

For Review Only

Supplementary Table 3: Step function model mixed effect model for CAMCOG-R per year of follow-up (left) with an interaction term on incident dementia (full model output)

	Complete follow-up				Last 3 yrs censored			
	Coef.	LL	UP	p	Coef.	LL	UP	p
Year 1	1.26	0.19	2.34	0.02	1.27	0.31	2.22	<0.01
Year 2	2.68	1.51	3.84	<0.01	2.68	1.65	3.71	<0.01
Year 3	1.76	0.49	3.03	<0.01	1.76	0.64	2.88	<0.01
Year 4	1.26	-0.13	2.64	0.08	1.26	0.03	2.48	0.04
Year 5	0.21	-1.27	1.69	0.78	0.21	-1.10	1.52	0.75
Year 6	0.25	-1.33	1.82	0.76	0.25	-1.15	1.64	0.73
Year 7	-1.09	-2.85	0.67	0.22	-1.09	-2.65	0.46	0.17
Year 8	-1.56	-3.76	0.65	0.17	-1.56	-3.51	0.39	0.12
Year 9	-3.62	-6.18	-1.06	<0.01	-3.62	-5.89	-1.35	<0.01
Year 10	-2.01	-5.27	1.25	0.23	-2.02	-4.90	0.87	0.17
Year 11	-4.17	-8.04	-0.30	0.04	-4.17	-7.60	-0.75	0.02
Year 12	-1.44	-5.31	2.43	0.47	-1.45	-4.88	1.98	0.41
Dementia	-6.04	-8.25	-3.83	<0.01	-2.13	-4.64	0.38	0.10
Dementia+1	-4.63	-6.64	-2.62	<0.01	-0.02	-2.63	2.59	0.99
Dementia+2	-4.78	-7.01	-2.54	<0.01	-0.15	-2.99	2.69	0.92
Dementia+3	-8.65	-11.08	-6.22	<0.01	-0.96	-4.08	2.15	0.55
Dementia+4	-8.02	-10.89	-5.15	<0.01	-2.86	-6.59	0.88	0.13
Dementia+5	-9.74	-12.75	-6.72	<0.01	-1.14	-5.37	3.09	0.60
Dementia+6	-13.95	-17.34	-10.56	<0.01	-5.88	-10.63	-1.13	0.02
Dementia+7	-12.47	-16.57	-8.37	<0.01	-4.72	-10.36	0.93	0.10
Dementia+8	-17.77	-22.58	-12.97	<0.01	-5.63	-13.30	2.04	0.15
Dementia+9	-14.30	-19.80	-8.80	<0.01	-5.07	-12.83	2.69	0.20
Dementia+10	-19.02	-25.73	-12.30	<0.01	-	-	-	-
Dementia+11	-19.53	-28.55	-10.50	<0.01	-	-	-	-
Dementia+12	-37.25	-46.27	-28.23	<0.01	-	-	-	-
Age	-2.47	-4.17	-0.77	<0.01	-3.00	-4.61	-1.39	<0.01
Gender	0.21	-1.50	1.92	0.81	0.09	-1.53	1.71	0.91
Constant	87.50	84.44	90.56	<0.01	87.96	85.07	90.84	<0.01

For Review Only

Supplementary Table 4: Step function model mixed effect model for MMSE per year of follow-up (left) with an interaction term on incident dementia (full model output)

	Complete follow-up				Last 3 yrs censored			
	Coef.	LL	UP	p	Coef.	LL	UP	p
Year 1	0.35	-0.07	0.77	0.107	1.27	0.31	2.22	0.009
Year 2	0.39	-0.06	0.85	0.092	2.68	1.65	3.71	0.000
Year 3	0.17	-0.32	0.67	0.495	1.76	0.64	2.88	0.002
Year 4	-0.13	-0.67	0.41	0.633	1.26	0.03	2.48	0.044
Year 5	-0.95	-1.53	-0.37	0.001	0.21	-1.10	1.52	0.753
Year 6	-0.66	-1.27	-0.04	0.037	0.25	-1.15	1.64	0.730
Year 7	-0.99	-1.67	-0.30	0.005	-1.09	-2.65	0.46	0.168
Year 8	-1.83	-2.69	-0.98	0.000	-1.56	-3.51	0.39	0.117
Year 9	-2.42	-3.40	-1.43	0.000	-3.62	-5.89	-1.35	0.002
Year 10	-1.57	-2.86	-0.29	0.016	-2.02	-4.90	0.87	0.171
Year 11	-2.21	-3.66	-0.76	0.003	-4.17	-7.60	-0.75	0.017
Year 12	-2.02	-3.47	-0.57	0.006	-1.45	-4.88	1.98	0.407
Dementia	-1.92	-2.65	-1.18	0.000	-2.13	-4.64	0.38	0.097
Dementia+1	-1.65	-2.44	-0.85	0.000	-0.02	-2.63	2.59	0.986
Dementia+2	-1.69	-2.56	-0.82	0.000	-0.15	-2.99	2.69	0.918
Dementia+3	-2.63	-3.58	-1.68	0.000	-0.96	-4.08	2.15	0.545
Dementia+4	-2.11	-3.23	-0.99	0.000	-2.86	-6.59	0.88	0.134
Dementia+5	-2.10	-3.28	-0.92	0.000	-1.14	-5.37	3.09	0.599
Dementia+6	-3.33	-4.66	-2.00	0.000	-5.88	-10.63	-1.13	0.015
Dementia+7	-3.82	-5.43	-2.21	0.000	-4.72	-10.36	0.93	0.101
Dementia+8	-4.66	-6.54	-2.77	0.000	-5.63	-13.30	2.04	0.150
Dementia+9	-3.85	-6.01	-1.69	0.000	-5.07	-12.83	2.69	0.200
Dementia+10	-5.46	-8.11	-2.82	0.000	-	-	-	-
Dementia+11	-4.32	-7.85	-0.80	0.016	-	-	-	-
Dementia+12	-7.52	-11.04	-3.99	0.000	-	-	-	-
Age	-0.57	-1.10	-0.05	0.033	-3.00	-4.61	-1.39	0.000
Gender	0.29	-0.24	0.82	0.283	0.09	-1.53	1.71	0.914
Constant	26.46	25.50	27.42	0.000	87.96	85.07	90.84	0.000

For Review Only

Supplementary Table 5: CAMCOG-R score for years of follow-up based on the step function stratified by length of follow-up

	Follow-up: 1 to 3 years				Follow-up: 4 to 6 years				Follow-up: 7 to 9 years				Follow-up: 10 to 12 years			
Years	Coef	LL	UL	P	Coef	LL	UL	P	Coef	LL	UL	P	Coef	LL	UL	P
1	-5.49	-8.55	-2.43	<0.01	1.77	-1.72	5.26	0.32	-1.00	-4.45	2.45	0.57	-0.75	-7.52	6.02	0.83
2	-4.42	-8.05	-0.78	0.02	1.20	-2.42	4.82	0.52	0.86	-2.60	4.31	0.63	2.11	-5.26	9.48	0.58
3	-15.24	-19.78	-10.70	<0.01	0.28	-3.28	3.83	0.88	-2.29	-5.74	1.17	0.20	0.00	-6.77	6.77	1.00
4	-	-	-	-	-4.96	-8.50	-1.42	<0.01	-3.00	-6.45	0.45	0.09	-4.00	-10.77	2.77	0.25
5	-	-	-	-	-10.00	-13.78	-6.21	<0.01	-4.43	-7.88	-0.98	0.01	-1.50	-8.27	5.27	0.66
6	-	-	-	-	-17.43	-22.29	-12.57	<0.01	-7.86	-11.31	-4.40	<0.01	-6.50	-13.27	0.27	0.06
7	-	-	-	-	-	-	-	-	-12.57	-16.02	-9.12	<0.01	-7.00	-13.77	-0.23	0.04
8	-	-	-	-	-	-	-	-	-24.13	-28.24	-20.02	<0.01	-8.00	-14.77	-1.23	0.02
9	-	-	-	-	-	-	-	-	-20.21	-25.54	-14.88	<0.01	-11.00	-17.77	-4.23	<0.01
10	-	-	-	-	-	-	-	-	-	-	-	-	-16.00	-22.77	-9.23	<0.01
11	-	-	-	-	-	-	-	-	-	-	-	-	-18.49	-26.91	-10.07	<0.01
12	-	-	-	-	-	-	-	-	-	-	-	-	-33.49	-41.91	-25.07	<0.01
age	1.36	-3.24	5.97	0.56	-4.69	-12.30	2.93	0.23	4.36	-0.87	9.59	0.10	-17.74	-32.63	-2.84	0.02
gender	1.79	-2.80	6.37	0.45	-0.38	-7.92	7.16	0.92	-4.88	-10.61	0.85	0.10	3.68	-11.26	18.62	0.63
Constant	75.59	68.09	83.09	<0.01	85.45	73.45	97.46	<0.01	93.83	85.59	102.07	<0.01	87.33	65.72	108.94	<0.01

For Review Only

Supplementary Table 6: Step-function model of CAMCOG-R change during follow-up, stratified by length of follow-up and censoring last 3 years

	Follow-up: 1 to 3 years				Follow-up: 4 to 6 years				Follow-up: 7 to 9 years				Follow-up: 10 to 12 years			
Years	Coef	LL	UL	P	Coef	LL	UL	P	Coef	LL	UL	P	Coef	LL	UL	P
1	-	-	-	-	2.50	-0.36	5.35	0.09	-1.00	-3.46	1.46	0.43	-0.75	-6.40	4.90	0.80
2	-	-	-	-	3.30	0.10	6.50	0.04	0.86	-1.60	3.32	0.50	1.53	-4.63	7.70	0.63
3	-	-	-	-	2.77	-1.30	6.85	0.18	-2.29	-4.75	0.18	0.07	0.00	-5.65	5.65	1.00
4	-	-	-	-	-	-	-	-	-3.00	-5.46	-0.54	0.02	-4.00	-9.65	1.65	0.17
5	-	-	-	-	-	-	-	-	-3.75	-6.70	-0.79	0.01	-1.50	-7.15	4.15	0.60
6	-	-	-	-	-	-	-	-	-8.43	-12.28	-4.58	<0.01	-6.50	-12.15	-0.85	0.02
7	-	-	-	-	-	-	-	-	-	-	-	-	-7.00	-12.65	-1.35	0.02
8	-	-	-	-	-	-	-	-	-	-	-	-	-8.06	-15.13	-0.99	0.03
9	-	-	-	-	-	-	-	-	-	-	-	-	-9.56	-16.63	-2.49	<0.01
10	-	-	-	-	-	-	-	-	-	-	-	-	-	-	-	-
11	-	-	-	-	-	-	-	-	-	-	-	-	-	-	-	-
12	-	-	-	-	-	-	-	-	-	-	-	-	-	-	-	-
age	0.00	0.00	0.00	<0.01	-2.63	-8.47	3.21	0.38	4.86	0.39	9.33	0.03	-18.59	-31.06	-6.12	<0.01
gender	0.00	0.00	0.00	<0.01	3.27	-2.51	9.05	0.27	-3.94	-8.84	0.96	0.12	5.51	-7.03	18.04	0.39
Constant	0.00	0.00	0.00	<0.01	78.69	69.51	87.87	<0.01	92.41	85.46	99.36	<0.01	85.26	67.15	103.37	<0.01

For Review Only

Supplementary Table 7: Step-function model of MMSE change during follow-up, stratified by length of follow-up

	Follow-up: 1 to 3 years				Follow-up: 4 to 6 years				Follow-up: 7 to 9 years				Follow-up: 10 to 12 years			
MMSE	Coef	LL	UL	p	Coef	LL	UL	p	Coef	LL	UL	p	Coef	LL	UL	p
1	-2.20	-3.37	-1.02	0.000	0.28	-0.94	1.51	0.649	0.57	-1.38	2.52	0.565	0.75	-1.80	3.30	0.564
2	-2.33	-3.70	-0.96	0.001	-0.47	-1.71	0.78	0.460	1.14	-0.80	3.09	0.250	0.42	-2.35	3.18	0.768
3	-5.88	-7.61	-4.16	0.000	-0.19	-1.44	1.06	0.764	0.57	-1.38	2.52	0.565	0.25	-2.30	2.80	0.847
4	-	-	-	-	-2.01	-3.25	-0.76	0.002	-0.29	-2.23	1.66	0.774	-0.75	-3.30	1.80	0.564
5	-	-	-	-	-3.58	-4.91	-2.26	0.000	-1.29	-3.23	0.66	0.196	1.25	-1.30	3.80	0.336
6	-	-	-	-	-4.94	-6.64	-3.24	0.000	-2.29	-4.23	-0.34	0.021	-0.75	-3.30	1.80	0.564
7	-	-	-	-	-	-	-	-	-4.43	-6.38	-2.48	0.000	-1.25	-3.80	1.30	0.336
8	-	-	-	-	-	-	-	-	-7.69	-9.99	-5.39	0.000	-1.75	-4.30	0.80	0.178
9	-	-	-	-	-	-	-	-	-4.76	-7.73	-1.79	0.002	-4.00	-6.55	-1.45	0.002
10	-	-	-	-	-	-	-	-	-	-	-	-	-4.75	-7.30	-2.20	0.000
11	-	-	-	-	-	-	-	-	-	-	-	-	-4.21	-7.38	-1.05	0.009
12	-	-	-	-	-	-	-	-	-	-	-	-	-7.21	-10.38	-4.05	0.000
Age	1.31	-0.20	2.82	0.089	-1.11	-3.52	1.31	0.368	1.59	0.25	2.92	0.020	-1.61	-6.19	2.98	0.492
Gender	1.38	-0.12	2.88	0.072	0.50	-1.89	2.89	0.684	-1.61	-3.08	-0.15	0.031	1.57	-3.04	6.17	0.505
Constant	21.59	19.12	24.07	0.000	25.62	21.80	29.44	0.000	27.82	25.43	30.21	0.000	24.19	17.46	30.92	0.000

For Review Only

Supplementary Table 8: Step-function model of MMSE change during follow-up, stratified by length of follow-up and censoring last 3 years

	Follow-up: 1 to 3 years				Follow-up: 4 to 6 years				Follow-up: 7 to 9 years				Follow-up: 10 to 12 years			
MMSE	Coef	LL	UL	p	Coef	LL	UL	p	Coef	LL	UL	p	Coef	LL	UL	p
1	-	-	-	-	0.45	-0.37	1.27	0.280	0.57	-1.03	2.17	0.485	0.75	-1.35	2.85	0.484
2	-	-	-	-	0.07	-0.82	0.97	0.870	1.14	-0.46	2.75	0.162	0.41	-1.88	2.71	0.723
3	-	-	-	-	1.04	-0.13	2.21	0.083	0.57	-1.03	2.17	0.485	0.25	-1.85	2.35	0.816
4	-	-	-	-	-	-	-	-	-0.29	-1.89	1.32	0.727	-0.75	-2.85	1.35	0.484
5	-	-	-	-	-	-	-	-	-1.56	-3.48	0.35	0.109	1.25	-0.85	3.35	0.244
6	-	-	-	-	-	-	-	-	-1.78	-4.26	0.70	0.160	-0.75	-2.85	1.35	0.484
7	-	-	-	-	-	-	-	-	-	-	-	-	-1.25	-3.35	0.85	0.244
8	-	-	-	-	-	-	-	-	-	-	-	-	-0.50	-3.12	2.13	0.710
9	-	-	-	-	-	-	-	-	-	-	-	-	-3.00	-5.62	-0.37	0.025
10	-	-	-	-	-	-	-	-	-	-	-	-	-	-	-	-
11	-	-	-	-	-	-	-	-	-	-	-	-	-	-	-	-
12	-	-	-	-	-	-	-	-	-	-	-	-	-	-	-	-
Age	-	-	-	-	-0.37	-2.46	1.72	0.728	2.01	0.42	3.60	0.013	-2.43	-6.77	1.91	0.272
Gender	-	-	-	-	1.29	-0.78	3.37	0.221	-1.71	-3.45	0.03	0.054	1.87	-2.49	6.23	0.401
Constant	-	-	-	-	23.98	20.71	27.25	0.000	27.77	25.17	30.37	0.000	24.02	17.70	30.34	0.000

For Review Only

Supplementary Table 9: Percentage of participants with a decline in CAMCOG-R score of 5 or more and 10 or more points in the year before diagnosis

CAMCOG-R decline	Length of follow-up			
5 or more points (%)	1-3 years	4-6 years	7-9 years	10-12 years
12 - 10 years	-	-	-	25
9 - 7 year	-	-	28.6	75
6 - 4 year	-	33.3	28.6	50
3 year – Diagnosis	69.8	94.1	100	100
10 or more points (%)				
12 - 10 years	-	-	-	0
9 - 7 year	-	-	0	25
6 - 4 year	-	5.6	14.3	0
3 year – Diagnosis	39.6	35.3	57.1	75

For Review Only

Supplementary Figure 1: Change in CAMCOG-R components (proportion of maximum score in the last three years of follow-up for the dementia group for the dementia group

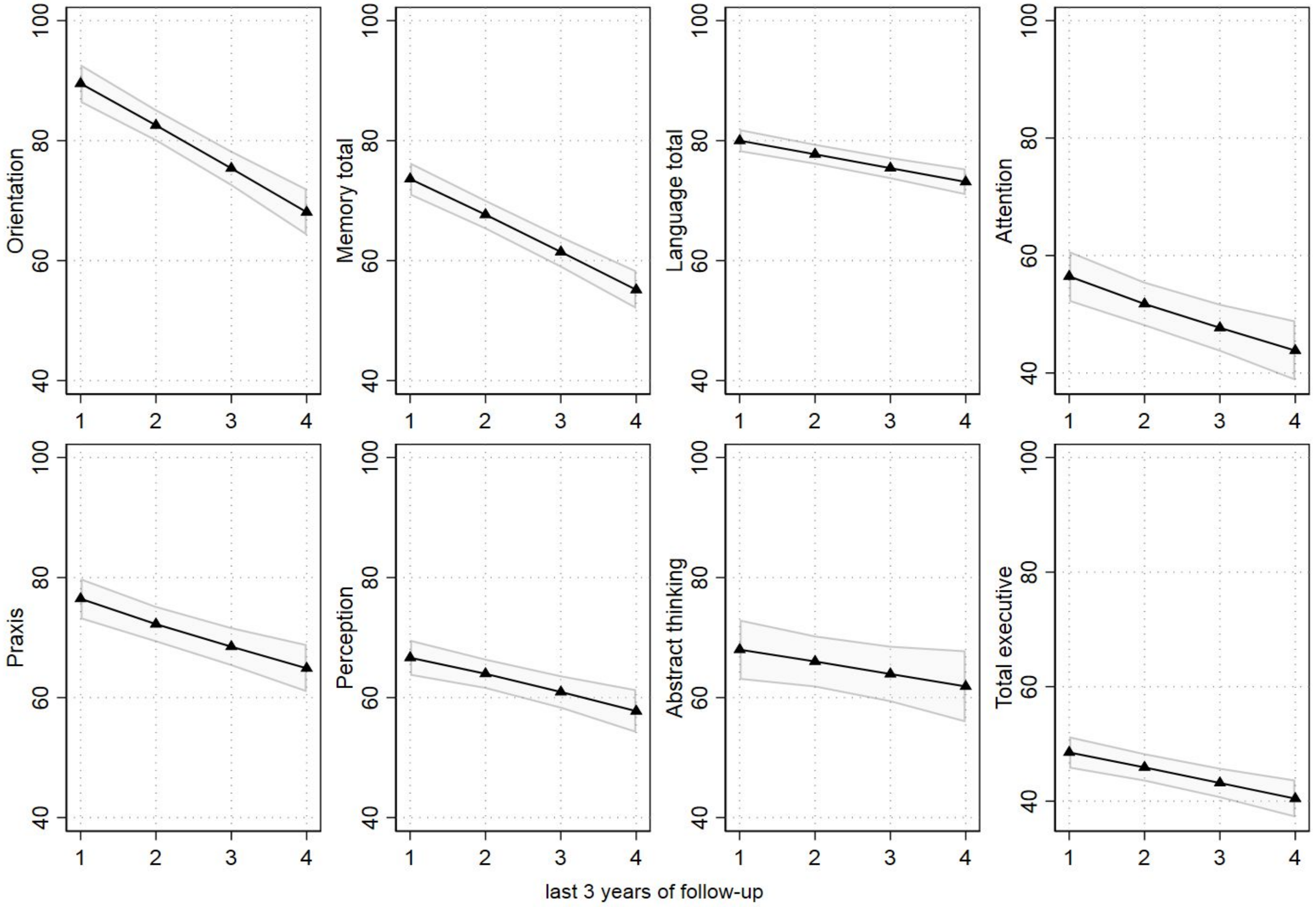

9

Supplementary Figure 2: Flow chart showing numbers screened, recruited and numbers of participants developing post-stroke dementia, including in the 4 follow-up categories.

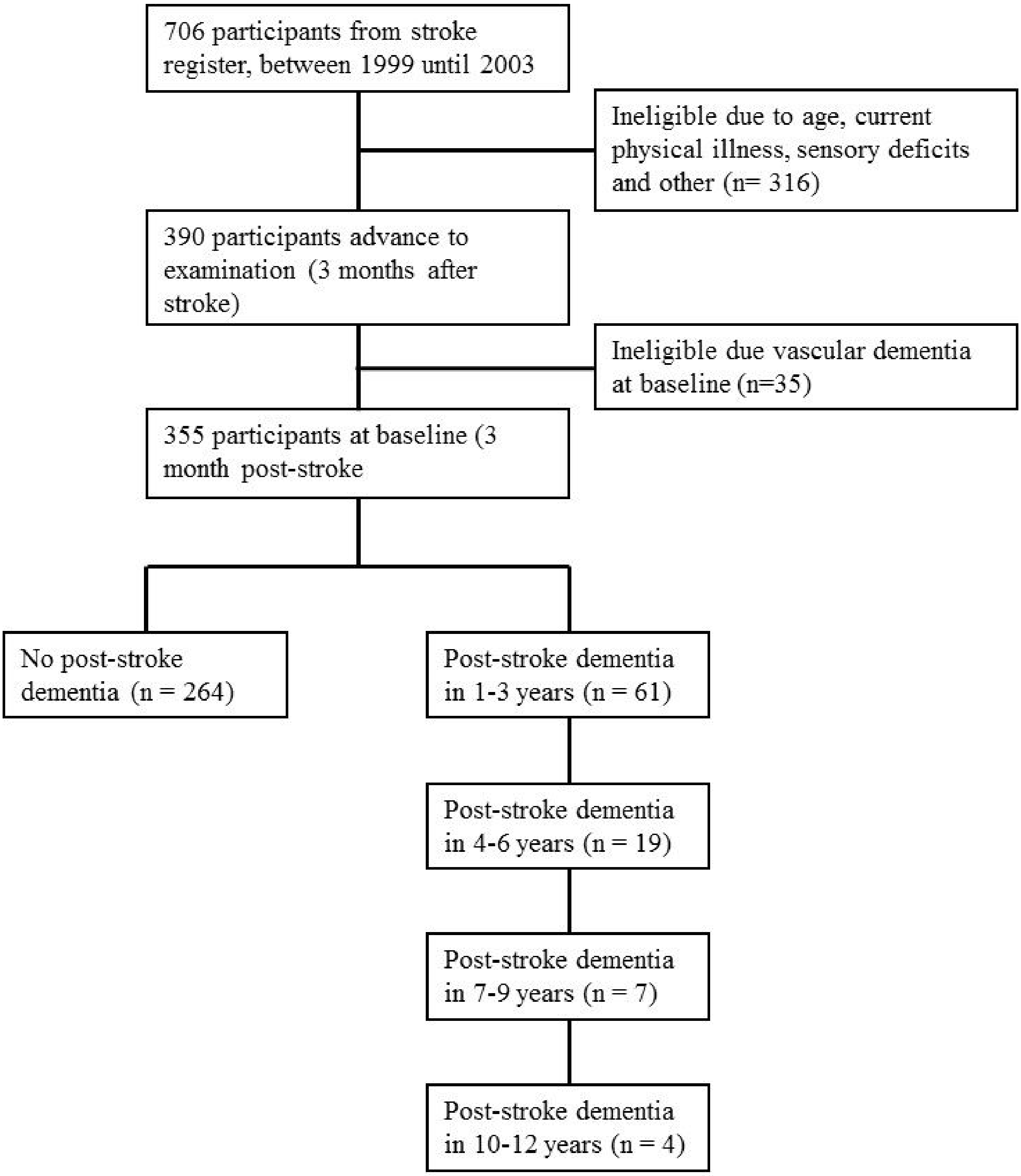

Supplement: fcac129_Supplementary_Data [file fcac129_supplementary_data.zip › Original Submission.pdf]
