# Supplementary material for: Trajectories of cognitive change following stroke: stepwise decline towards dementia in the elderly
Source: Brain Commun. 2022 May 24;4(3):fcac129. doi: 10.1093/braincomms/fcac129 (PMC9161377; doi:10.1093/braincomms/fcac129)

Supplemental materials

# Tables

Supplementary Table 1: Linear mixed effect model for CAMCOG-R per year of follow-up (left) with an interaction term on incident dementia (left)

|  | a) Simple model | | b) interaction on dementia | | c) 3 years censored | |
| --- | --- | --- | --- | --- | --- | --- |
|  | coef. | CI 95% | coef. | CI 95% | coef. | CI 95% |
| Year | -0.68* | -0.82:-0.54 | -0.25* | -0.38:-0.12 | -0.25* | -0.38:-0.12 |
| Dementia | - | -:- | -7.1* | -9.16:-5.05 | -2.11 | -4.52:0.31 |
| Dementia + Year | - | -:- | -1.91* | -2.23:-1.59 | -0.49* | -0.97:0.00 |
| Age | -2.07* | -4.03:-0.11 | -2.43* | -4.16:-0.7 | -3.05* | -4.70:-1.39 |
| Gender | 1.61 | -0.36:3.57 | 0.38 | -1.36:2.13 | 0.23 | -1.43:1.90 |
| Constant | 84.35* | 80.97:87.72 | 88.42* | 85.31:91.52 | 88.96* | 86.02:91.90 |

* P-values <0.05

Supplementary Table 2: Linear mixed effect model for MMSE per year of follow-up (left) with an interaction term on incident dementia (left)

|  | a) Simple model | | b) interaction on dementia | | c) 3 years censored | |
| --- | --- | --- | --- | --- | --- | --- |
|  | coef. | CI 95% | coef. | CI 95% | coef. | CI 95% |
| Year | -0.32* | -0.37:-0.26 | -0.21* | -0.26:-0.15 | -0.20* | -0.25:-0.15 |
| Dementia |  |  | -2.46* | -3.11:-1.81 | -0.74 | -1.48:0.01 |
| Dementia + Year |  |  | -0.46* | -0.58:-0.34 | 0.03 | -0.16:0.21 |
| Age | -0.44 | -1.05:0.17 | -0.57* | -1.10:-0.03 | -0.83* | -1.33:-0.34 |
| Gender | 0.73* | 0.12:1.34 | 0.33 | -0.21:0.87 | 0.22 | -0.27:0.72 |
| Constant | 25.40* | 24.34:26.45 | 26.76* | 25.80:27.73 | 27.08* | 26.20:27.95 |

* P-values <0.05

Supplementary Table 3: Step function model mixed effect model for CAMCOG-R per year of follow-up (left) with an interaction term on incident dementia (full model output)

|  | Complete follow-up | | | | Last 3 yrs censored | | | |
| --- | --- | --- | --- | --- | --- | --- | --- | --- |
|  | Coef. | LL | UP | p | Coef. | LL | UP | p |
| Year 1 | 1.26 | 0.19 | 2.34 | 0.02 | 1.27 | 0.31 | 2.22 | <0.01 |
| Year 2 | 2.68 | 1.51 | 3.84 | <0.01 | 2.68 | 1.65 | 3.71 | <0.01 |
| Year 3 | 1.76 | 0.49 | 3.03 | <0.01 | 1.76 | 0.64 | 2.88 | <0.01 |
| Year 4 | 1.26 | -0.13 | 2.64 | 0.08 | 1.26 | 0.03 | 2.48 | 0.04 |
| Year 5 | 0.21 | -1.27 | 1.69 | 0.78 | 0.21 | -1.10 | 1.52 | 0.75 |
| Year 6 | 0.25 | -1.33 | 1.82 | 0.76 | 0.25 | -1.15 | 1.64 | 0.73 |
| Year 7 | -1.09 | -2.85 | 0.67 | 0.22 | -1.09 | -2.65 | 0.46 | 0.17 |
| Year 8 | -1.56 | -3.76 | 0.65 | 0.17 | -1.56 | -3.51 | 0.39 | 0.12 |
| Year 9 | -3.62 | -6.18 | -1.06 | <0.01 | -3.62 | -5.89 | -1.35 | <0.01 |
| Year 10 | -2.01 | -5.27 | 1.25 | 0.23 | -2.02 | -4.90 | 0.87 | 0.17 |
| Year 11 | -4.17 | -8.04 | -0.30 | 0.04 | -4.17 | -7.60 | -0.75 | 0.02 |
| Year 12 | -1.44 | -5.31 | 2.43 | 0.47 | -1.45 | -4.88 | 1.98 | 0.41 |
| Dementia | -6.04 | -8.25 | -3.83 | <0.01 | -2.13 | -4.64 | 0.38 | 0.10 |
| Dementia+1 | -4.63 | -6.64 | -2.62 | <0.01 | -0.02 | -2.63 | 2.59 | 0.99 |
| Dementia+2 | -4.78 | -7.01 | -2.54 | <0.01 | -0.15 | -2.99 | 2.69 | 0.92 |
| Dementia+3 | -8.65 | -11.08 | -6.22 | <0.01 | -0.96 | -4.08 | 2.15 | 0.55 |
| Dementia+4 | -8.02 | -10.89 | -5.15 | <0.01 | -2.86 | -6.59 | 0.88 | 0.13 |
| Dementia+5 | -9.74 | -12.75 | -6.72 | <0.01 | -1.14 | -5.37 | 3.09 | 0.60 |
| Dementia+6 | -13.95 | -17.34 | -10.56 | <0.01 | -5.88 | -10.63 | -1.13 | 0.02 |
| Dementia+7 | -12.47 | -16.57 | -8.37 | <0.01 | -4.72 | -10.36 | 0.93 | 0.10 |
| Dementia+8 | -17.77 | -22.58 | -12.97 | <0.01 | -5.63 | -13.30 | 2.04 | 0.15 |
| Dementia+9 | -14.30 | -19.80 | -8.80 | <0.01 | -5.07 | -12.83 | 2.69 | 0.20 |
| Dementia+10 | -19.02 | -25.73 | -12.30 | <0.01 | - | - | - | - |
| Dementia+11 | -19.53 | -28.55 | -10.50 | <0.01 | - | - | - | - |
| Dementia+12 | -37.25 | -46.27 | -28.23 | <0.01 | - | - | - | - |
| Age | -2.47 | -4.17 | -0.77 | <0.01 | -3.00 | -4.61 | -1.39 | <0.01 |
| Gender | 0.21 | -1.50 | 1.92 | 0.81 | 0.09 | -1.53 | 1.71 | 0.91 |
| Constant | 87.50 | 84.44 | 90.56 | <0.01 | 87.96 | 85.07 | 90.84 | <0.01 |

Supplementary Table 4: Step function model mixed effect model for MMSE per year of follow-up (left) with an interaction term on incident dementia (full model output)

|  | Complete follow-up | | | | Last 3 yrs censored | | | |
| --- | --- | --- | --- | --- | --- | --- | --- | --- |
|  | Coef. | LL | UP | p | Coef. | LL | UP | p |
| Year 1 | 0.35 | -0.07 | 0.77 | 0.107 | 1.27 | 0.31 | 2.22 | 0.009 |
| Year 2 | 0.39 | -0.06 | 0.85 | 0.092 | 2.68 | 1.65 | 3.71 | 0.000 |
| Year 3 | 0.17 | -0.32 | 0.67 | 0.495 | 1.76 | 0.64 | 2.88 | 0.002 |
| Year 4 | -0.13 | -0.67 | 0.41 | 0.633 | 1.26 | 0.03 | 2.48 | 0.044 |
| Year 5 | -0.95 | -1.53 | -0.37 | 0.001 | 0.21 | -1.10 | 1.52 | 0.753 |
| Year 6 | -0.66 | -1.27 | -0.04 | 0.037 | 0.25 | -1.15 | 1.64 | 0.730 |
| Year 7 | -0.99 | -1.67 | -0.30 | 0.005 | -1.09 | -2.65 | 0.46 | 0.168 |
| Year 8 | -1.83 | -2.69 | -0.98 | 0.000 | -1.56 | -3.51 | 0.39 | 0.117 |
| Year 9 | -2.42 | -3.40 | -1.43 | 0.000 | -3.62 | -5.89 | -1.35 | 0.002 |
| Year 10 | -1.57 | -2.86 | -0.29 | 0.016 | -2.02 | -4.90 | 0.87 | 0.171 |
| Year 11 | -2.21 | -3.66 | -0.76 | 0.003 | -4.17 | -7.60 | -0.75 | 0.017 |
| Year 12 | -2.02 | -3.47 | -0.57 | 0.006 | -1.45 | -4.88 | 1.98 | 0.407 |
| Dementia | -1.92 | -2.65 | -1.18 | 0.000 | -2.13 | -4.64 | 0.38 | 0.097 |
| Dementia+1 | -1.65 | -2.44 | -0.85 | 0.000 | -0.02 | -2.63 | 2.59 | 0.986 |
| Dementia+2 | -1.69 | -2.56 | -0.82 | 0.000 | -0.15 | -2.99 | 2.69 | 0.918 |
| Dementia+3 | -2.63 | -3.58 | -1.68 | 0.000 | -0.96 | -4.08 | 2.15 | 0.545 |
| Dementia+4 | -2.11 | -3.23 | -0.99 | 0.000 | -2.86 | -6.59 | 0.88 | 0.134 |
| Dementia+5 | -2.10 | -3.28 | -0.92 | 0.000 | -1.14 | -5.37 | 3.09 | 0.599 |
| Dementia+6 | -3.33 | -4.66 | -2.00 | 0.000 | -5.88 | -10.63 | -1.13 | 0.015 |
| Dementia+7 | -3.82 | -5.43 | -2.21 | 0.000 | -4.72 | -10.36 | 0.93 | 0.101 |
| Dementia+8 | -4.66 | -6.54 | -2.77 | 0.000 | -5.63 | -13.30 | 2.04 | 0.150 |
| Dementia+9 | -3.85 | -6.01 | -1.69 | 0.000 | -5.07 | -12.83 | 2.69 | 0.200 |
| Dementia+10 | -5.46 | -8.11 | -2.82 | 0.000 | - | - | - | - |
| Dementia+11 | -4.32 | -7.85 | -0.80 | 0.016 | - | - | - | - |
| Dementia+12 | -7.52 | -11.04 | -3.99 | 0.000 | - | - | - | - |
| Age | -0.57 | -1.10 | -0.05 | 0.033 | -3.00 | -4.61 | -1.39 | 0.000 |
| Gender | 0.29 | -0.24 | 0.82 | 0.283 | 0.09 | -1.53 | 1.71 | 0.914 |
| Constant | 26.46 | 25.50 | 27.42 | 0.000 | 87.96 | 85.07 | 90.84 | 0.000 |

Supplementary Table 5: CAMCOG-R score for years of follow-up based on the step function stratified by length of follow-up

|  | Follow-up: 1 to 3 years | | | | Follow-up: 4 to 6 years | | | | Follow-up: 7 to 9 years | | | | Follow-up: 10 to 12 years | | | |
| --- | --- | --- | --- | --- | --- | --- | --- | --- | --- | --- | --- | --- | --- | --- | --- | --- |
| Years | Coef | LL | UL | P | Coef | LL | UL | P | Coef | LL | UL | P | Coef | LL | UL | P |
| 1 | -5.49 | -8.55 | -2.43 | <0.01 | 1.77 | -1.72 | 5.26 | 0.32 | -1.00 | -4.45 | 2.45 | 0.57 | -0.75 | -7.52 | 6.02 | 0.83 |
| 2 | -4.42 | -8.05 | -0.78 | 0.02 | 1.20 | -2.42 | 4.82 | 0.52 | 0.86 | -2.60 | 4.31 | 0.63 | 2.11 | -5.26 | 9.48 | 0.58 |
| 3 | -15.24 | -19.78 | -10.70 | <0.01 | 0.28 | -3.28 | 3.83 | 0.88 | -2.29 | -5.74 | 1.17 | 0.20 | 0.00 | -6.77 | 6.77 | 1.00 |
| 4 | - | - | - | - | -4.96 | -8.50 | -1.42 | <0.01 | -3.00 | -6.45 | 0.45 | 0.09 | -4.00 | -10.77 | 2.77 | 0.25 |
| 5 | - | - | - | - | -10.00 | -13.78 | -6.21 | <0.01 | -4.43 | -7.88 | -0.98 | 0.01 | -1.50 | -8.27 | 5.27 | 0.66 |
| 6 | - | - | - | - | -17.43 | -22.29 | -12.57 | <0.01 | -7.86 | -11.31 | -4.40 | <0.01 | -6.50 | -13.27 | 0.27 | 0.06 |
| 7 | - | - | - | - | - | - | - | - | -12.57 | -16.02 | -9.12 | <0.01 | -7.00 | -13.77 | -0.23 | 0.04 |
| 8 | - | - | - | - | - | - | - | - | -24.13 | -28.24 | -20.02 | <0.01 | -8.00 | -14.77 | -1.23 | 0.02 |
| 9 | - | - | - | - | - | - | - | - | -20.21 | -25.54 | -14.88 | <0.01 | -11.00 | -17.77 | -4.23 | <0.01 |
| 10 | - | - | - | - | - | - | - | - | - | - | - | - | -16.00 | -22.77 | -9.23 | <0.01 |
| 11 | - | - | - | - | - | - | - | - | - | - | - | - | -18.49 | -26.91 | -10.07 | <0.01 |
| 12 | - | - | - | - | - | - | - | - | - | - | - | - | -33.49 | -41.91 | -25.07 | <0.01 |
| age | 1.36 | -3.24 | 5.97 | 0.56 | -4.69 | -12.30 | 2.93 | 0.23 | 4.36 | -0.87 | 9.59 | 0.10 | -17.74 | -32.63 | -2.84 | 0.02 |
| gender | 1.79 | -2.80 | 6.37 | 0.45 | -0.38 | -7.92 | 7.16 | 0.92 | -4.88 | -10.61 | 0.85 | 0.10 | 3.68 | -11.26 | 18.62 | 0.63 |
| Constant | 75.59 | 68.09 | 83.09 | <0.01 | 85.45 | 73.45 | 97.46 | <0.01 | 93.83 | 85.59 | 102.07 | <0.01 | 87.33 | 65.72 | 108.94 | <0.01 |

Supplementary Table 6: Step-function model of CAMCOG-R change during follow-up, stratified by length of follow-up and censoring last 3 years

|  | Follow-up: 1 to 3 years | | | | Follow-up: 4 to 6 years | | | | Follow-up: 7 to 9 years | | | | Follow-up: 10 to 12 years | | | |
| --- | --- | --- | --- | --- | --- | --- | --- | --- | --- | --- | --- | --- | --- | --- | --- | --- |
| Years | Coef | LL | UL | P | Coef | LL | UL | P | Coef | LL | UL | P | Coef | LL | UL | P |
| 1 | - | - | - | - | 2.50 | -0.36 | 5.35 | 0.09 | -1.00 | -3.46 | 1.46 | 0.43 | -0.75 | -6.40 | 4.90 | 0.80 |
| 2 | - | - | - | - | 3.30 | 0.10 | 6.50 | 0.04 | 0.86 | -1.60 | 3.32 | 0.50 | 1.53 | -4.63 | 7.70 | 0.63 |
| 3 | - | - | - | - | 2.77 | -1.30 | 6.85 | 0.18 | -2.29 | -4.75 | 0.18 | 0.07 | 0.00 | -5.65 | 5.65 | 1.00 |
| 4 | - | - | - | - | - | - | - | - | -3.00 | -5.46 | -0.54 | 0.02 | -4.00 | -9.65 | 1.65 | 0.17 |
| 5 | - | - | - | - | - | - | - | - | -3.75 | -6.70 | -0.79 | 0.01 | -1.50 | -7.15 | 4.15 | 0.60 |
| 6 | - | - | - | - | - | - | - | - | -8.43 | -12.28 | -4.58 | <0.01 | -6.50 | -12.15 | -0.85 | 0.02 |
| 7 | - | - | - | - | - | - | - | - | - | - | - | - | -7.00 | -12.65 | -1.35 | 0.02 |
| 8 | - | - | - | - | - | - | - | - | - | - | - | - | -8.06 | -15.13 | -0.99 | 0.03 |
| 9 | - | - | - | - | - | - | - | - | - | - | - | - | -9.56 | -16.63 | -2.49 | <0.01 |
| 10 | - | - | - | - | - | - | - | - | - | - | - | - | - | - | - | - |
| 11 | - | - | - | - | - | - | - | - | - | - | - | - | - | - | - | - |
| 12 | - | - | - | - | - | - | - | - | - | - | - | - | - | - | - | - |
| age | 0.00 | 0.00 | 0.00 | <0.01 | -2.63 | -8.47 | 3.21 | 0.38 | 4.86 | 0.39 | 9.33 | 0.03 | -18.59 | -31.06 | -6.12 | <0.01 |
| gender | 0.00 | 0.00 | 0.00 | <0.01 | 3.27 | -2.51 | 9.05 | 0.27 | -3.94 | -8.84 | 0.96 | 0.12 | 5.51 | -7.03 | 18.04 | 0.39 |
| Constant | 0.00 | 0.00 | 0.00 | <0.01 | 78.69 | 69.51 | 87.87 | <0.01 | 92.41 | 85.46 | 99.36 | <0.01 | 85.26 | 67.15 | 103.37 | <0.01 |

Supplementary Table 7: Step-function model of MMSE change during follow-up, stratified by length of follow-up

|  | Follow-up: 1 to 3 years | | | | Follow-up: 4 to 6 years | | | | Follow-up: 7 to 9 years | | | | Follow-up: 10 to 12 years | | | |
| --- | --- | --- | --- | --- | --- | --- | --- | --- | --- | --- | --- | --- | --- | --- | --- | --- |
| MMSE | Coef | LL | UL | p | Coef | LL | UL | p | Coef | LL | UL | p | Coef | LL | UL | p |
| 1 | -2.20 | -3.37 | -1.02 | 0.000 | 0.28 | -0.94 | 1.51 | 0.649 | 0.57 | -1.38 | 2.52 | 0.565 | 0.75 | -1.80 | 3.30 | 0.564 |
| 2 | -2.33 | -3.70 | -0.96 | 0.001 | -0.47 | -1.71 | 0.78 | 0.460 | 1.14 | -0.80 | 3.09 | 0.250 | 0.42 | -2.35 | 3.18 | 0.768 |
| 3 | -5.88 | -7.61 | -4.16 | 0.000 | -0.19 | -1.44 | 1.06 | 0.764 | 0.57 | -1.38 | 2.52 | 0.565 | 0.25 | -2.30 | 2.80 | 0.847 |
| 4 | - | - | - | - | -2.01 | -3.25 | -0.76 | 0.002 | -0.29 | -2.23 | 1.66 | 0.774 | -0.75 | -3.30 | 1.80 | 0.564 |
| 5 | - | - | - | - | -3.58 | -4.91 | -2.26 | 0.000 | -1.29 | -3.23 | 0.66 | 0.196 | 1.25 | -1.30 | 3.80 | 0.336 |
| 6 | - | - | - | - | -4.94 | -6.64 | -3.24 | 0.000 | -2.29 | -4.23 | -0.34 | 0.021 | -0.75 | -3.30 | 1.80 | 0.564 |
| 7 | - | - | - | - | - | - | - | - | -4.43 | -6.38 | -2.48 | 0.000 | -1.25 | -3.80 | 1.30 | 0.336 |
| 8 | - | - | - | - | - | - | - | - | -7.69 | -9.99 | -5.39 | 0.000 | -1.75 | -4.30 | 0.80 | 0.178 |
| 9 | - | - | - | - | - | - | - | - | -4.76 | -7.73 | -1.79 | 0.002 | -4.00 | -6.55 | -1.45 | 0.002 |
| 10 | - | - | - | - | - | - | - | - | - | - | - | - | -4.75 | -7.30 | -2.20 | 0.000 |
| 11 | - | - | - | - | - | - | - | - | - | - | - | - | -4.21 | -7.38 | -1.05 | 0.009 |
| 12 | - | - | - | - | - | - | - | - | - | - | - | - | -7.21 | -10.38 | -4.05 | 0.000 |
| Age | 1.31 | -0.20 | 2.82 | 0.089 | -1.11 | -3.52 | 1.31 | 0.368 | 1.59 | 0.25 | 2.92 | 0.020 | -1.61 | -6.19 | 2.98 | 0.492 |
| Gender | 1.38 | -0.12 | 2.88 | 0.072 | 0.50 | -1.89 | 2.89 | 0.684 | -1.61 | -3.08 | -0.15 | 0.031 | 1.57 | -3.04 | 6.17 | 0.505 |
| Constant | 21.59 | 19.12 | 24.07 | 0.000 | 25.62 | 21.80 | 29.44 | 0.000 | 27.82 | 25.43 | 30.21 | 0.000 | 24.19 | 17.46 | 30.92 | 0.000 |

Supplementary Table 8: Step-function model of MMSE change during follow-up, stratified by length of follow-up and censoring last 3 years

|  | Follow-up: 1 to 3 years | | | | Follow-up: 4 to 6 years | | | | Follow-up: 7 to 9 years | | | | Follow-up: 10 to 12 years | | | |
| --- | --- | --- | --- | --- | --- | --- | --- | --- | --- | --- | --- | --- | --- | --- | --- | --- |
| MMSE | Coef | LL | UL | p | Coef | LL | UL | p | Coef | LL | UL | p | Coef | LL | UL | p |
| 1 | - | - | - | - | 0.45 | -0.37 | 1.27 | 0.280 | 0.57 | -1.03 | 2.17 | 0.485 | 0.75 | -1.35 | 2.85 | 0.484 |
| 2 | - | - | - | - | 0.07 | -0.82 | 0.97 | 0.870 | 1.14 | -0.46 | 2.75 | 0.162 | 0.41 | -1.88 | 2.71 | 0.723 |
| 3 | - | - | - | - | 1.04 | -0.13 | 2.21 | 0.083 | 0.57 | -1.03 | 2.17 | 0.485 | 0.25 | -1.85 | 2.35 | 0.816 |
| 4 | - | - | - | - | - | - | - | - | -0.29 | -1.89 | 1.32 | 0.727 | -0.75 | -2.85 | 1.35 | 0.484 |
| 5 | - | - | - | - | - | - | - | - | -1.56 | -3.48 | 0.35 | 0.109 | 1.25 | -0.85 | 3.35 | 0.244 |
| 6 | - | - | - | - | - | - | - | - | -1.78 | -4.26 | 0.70 | 0.160 | -0.75 | -2.85 | 1.35 | 0.484 |
| 7 | - | - | - | - | - | - | - | - | - | - | - | - | -1.25 | -3.35 | 0.85 | 0.244 |
| 8 | - | - | - | - | - | - | - | - | - | - | - | - | -0.50 | -3.12 | 2.13 | 0.710 |
| 9 | - | - | - | - | - | - | - | - | - | - | - | - | -3.00 | -5.62 | -0.37 | 0.025 |
| 10 | - | - | - | - | - | - | - | - | - | - | - | - | - | - | - | - |
| 11 | - | - | - | - | - | - | - | - | - | - | - | - | - | - | - | - |
| 12 | - | - | - | - | - | - | - | - | - | - | - | - | - | - | - | - |
| Age | - | - | - | - | -0.37 | -2.46 | 1.72 | 0.728 | 2.01 | 0.42 | 3.60 | 0.013 | -2.43 | -6.77 | 1.91 | 0.272 |
| Gender | - | - | - | - | 1.29 | -0.78 | 3.37 | 0.221 | -1.71 | -3.45 | 0.03 | 0.054 | 1.87 | -2.49 | 6.23 | 0.401 |
| Constant | - | - | - | - | 23.98 | 20.71 | 27.25 | 0.000 | 27.77 | 25.17 | 30.37 | 0.000 | 24.02 | 17.70 | 30.34 | 0.000 |

Supplementary Table 9: Percentage of participants with a decline in CAMCOG-R score of 5 or more and 10 or more points in the year before diagnosis

| CAMCOG-R decline | Length of follow-up | | | |
| --- | --- | --- | --- | --- |
| 5 or more points (%) | 1-3 years | 4-6 years | 7-9 years | 10-12 years |
| 12 - 10 years | - | - | - | 25 |
| 9 - 7 year | - | - | 28.6 | 75 |
| 6 - 4 year | - | 33.3 | 28.6 | 50 |
| 3 year – Diagnosis | 69.8 | 94.1 | 100 | 100 |
| 10 or more points (%) |  |  |  |  |
| 12 - 10 years | - | - | - | 0 |
| 9 - 7 year | - | - | 0 | 25 |
| 6 - 4 year | - | 5.6 | 14.3 | 0 |
| 3 year – Diagnosis | 39.6 | 35.3 | 57.1 | 75 |

Supplementary Figure 1: Change in CAMCOG-R components (proportion of maximum score in the last three years of follow-up for the dementia group for the dementia group


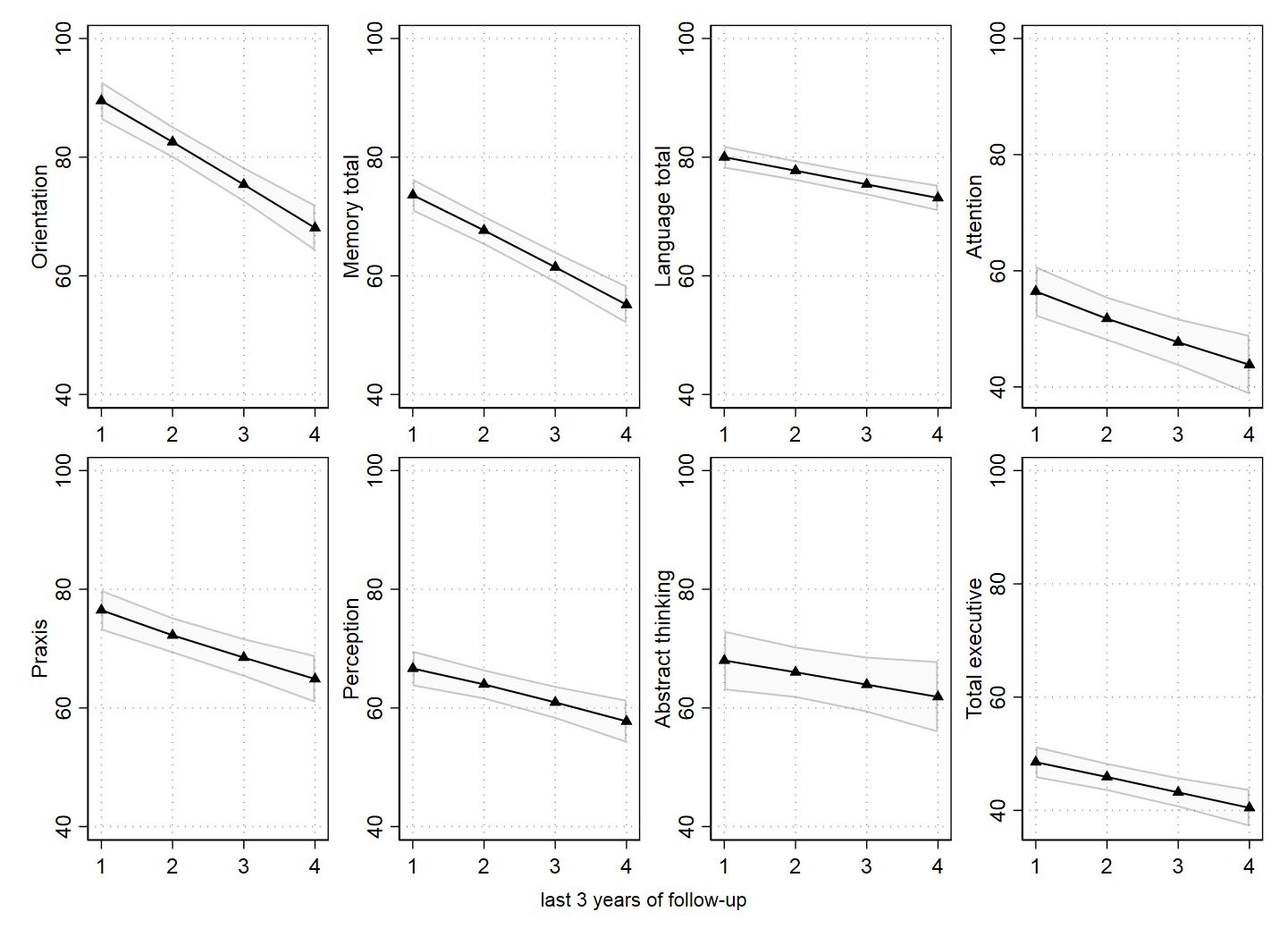


Supplementary Figure 2: Flow chart showing numbers screened, recruited and numbers of participants developing post-stroke dementia, including in the 4 follow-up categories.


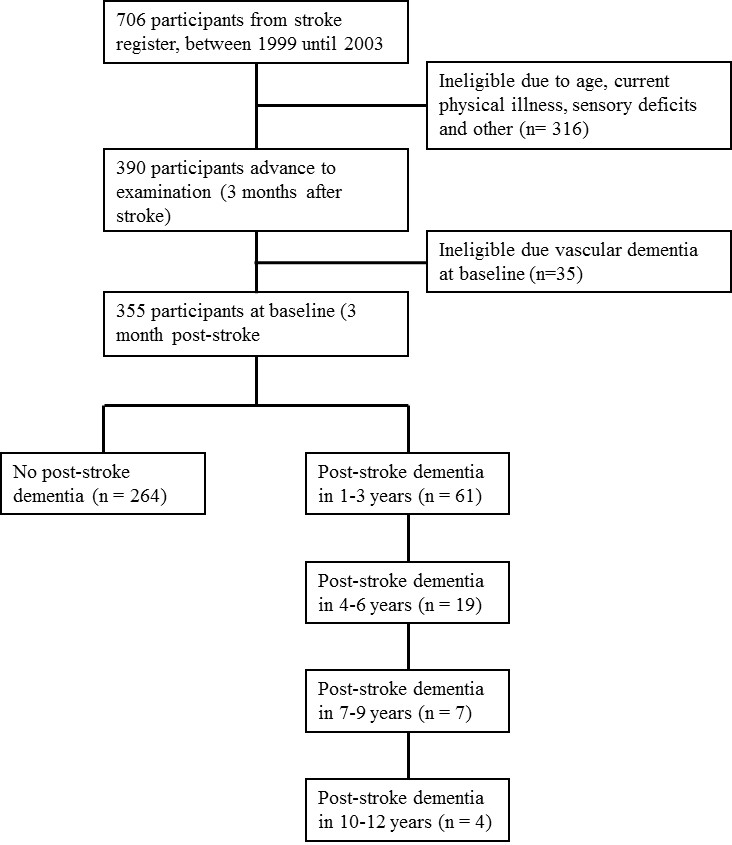

Supplement: fcac129_Supplementary_Data [file fcac129_supplementary_data.zip › Supplementary Document.docx]
