# Supplementary material for: Trajectories of cognitive change following stroke: stepwise decline towards dementia in the elderly
Source: Brain Commun. 2022 May 24;4(3):fcac129. doi: 10.1093/braincomms/fcac129 (PMC9161377; doi:10.1093/braincomms/fcac129)
Supplement: fcac129_Supplementary_Data [file Original Submission - Author's Response.pdf › Original Submission - Author's Response.pdf]

Trajectories of cognitive change following stroke: stepwise decline towards dementia in the elderly, *J Delgado et al, Brain Communications, 2022*

Replies to reviewers

#	Comment	Reply	Page
Reviewer 1			
1	The current study investigates trajectories of cognitive change/ incident dementia after stroke. This is a well-organized and well written research, adding interesting insights into the field of cognitive aging/ dementia. Big strength the length of the follow-up and the relatively big sample size	We thank the reviewers for this comment.	
2	Did you include MCI at baseline? If no, please add this info in the Methods section, if yes, please clarify and probably report results with/out the MCI group	Thank you for bringing our attention to this important point. We did not include the widely recognised MCI criteria but had previously reported that CIND (cognitive impairment no dementia) cases at baseline (Allan et al, 2011). We have now clarified this point in the Methods section	Page 6, line 6-8
3	Cognitive outcomes: did you transform to z-scores?	Thank you for this comment. We have clarified in the text that all participants in the study had both CAMCOG-R and MMSE scores recorded at baseline. We have also clarified that in separate analysis on the same sample which had CAMCOG-R and MMSE scores. The two scores systems were not combined for any analyses.<br><br>We have now included the summary statistic for z-scores for CAMCOG-R and MMSE for the whole sample at baseline and end of follow-up, then reported the means and SDs (p-value from Kruskal–Wallis test) for dementia and no dementia in Table 1.	Page 5 Line 20 Page 6 Line 14 Table 1
4	Why both CAMCOG and MMSE were used? MMSE is quite short and is more of a limitation to make research conclusions regarding cognition based on this	Thanks. As MMSE is used widely rather than the CAMCOG battery we also wanted to explore the utility of MMSE. We agree MMSE is indicative but not entirely reliable. However, its addition confirms our general observations. Our conclusions are largely based on the CAMCOG scores but felt that we should make a statement about MMSE.	Page 6, line 6-8
5	Make clear the cognitive domains and the analyses regarding them, both in the Methods and the Results sections	We have acted on this comment and clarified this in the methods and results section.	Page 5, line 19 Page 6, line 14

6	Statistical analysis: did you use any covariates? I would recommend at least using age, sex, and education (probably BMI too) (it is only mentioned at the line plot, but which ones where used, in which models etc.? Also, confused with the Risk factors, are they used as a covariate?)	Thanks. We have now acted on this comment and edited the methods section to clarify the analyses.<br><br>Analysis on longitudinal change measured using mixed effect models on CAMCOG-R and then on MMSE were adjusted for age (<80 and ≥80 years) and sex. We regret, the body mass index (or alternative measures of body composition), education or measures of deprivation were not obtained at baseline.<br><br>Univariate analysis for potential risk factors was adjusted for continuous age and sex. The multivariable model includes - in addition to continuous age and gender - OCSP classification, previous stroke, CIND, APOE ε4, Hypertension, Myocardial Infarction, Ischaemic Heart Disease, Type 2 Diabetes, Atrial Fibrillation, Hypercholesterolemia, Smoking history.<br><br>Previous disabling stroke was not included in either the univariate or multivariate analyses, as 0 cases of previous disabling stroke were identified in the group developing dementia after 3 or more years.	Page 6 Line 20. Page 8 line 23
7	What type of dementia was seen in the follow-up? Percentages of vascular vs other?	This is a good point. We have reported previously, that over 75% of the cases fit into VaD criteria as defined by Skrobot et al, 2018 and others. Our subsequent analyses and pathological findings suggest even more proportions of cases >78% can be classed as VaD. The rest were mixed with enough diagnostic AD type of pathology (Kalaria et al, in preparation).<br><br>The text has been edited to clarify this point: *We previously reported that >75% of the post-mortem cases fit into VaD criteria.3,12,24 Our subsequent analyses and pathological findings suggest even more cases can be classed as VaD. The rest of the cases were mixed with diagnostic AD type of pathology (RNK et al, unpublished data).*	Page 13 Line 23
8	do you think is that a matter of Cognitive Reserve or Brain Reserve? Please advise Stern et al., 2020 (Whitepaper) and expand more.	Thank you. We agree it is an important distinction to make but probably both these play a role in stability and outcome after stroke.<br>We have now incorporated these aspects into the discussion: *"Our findings are in accord with the concepts of both cognitive and brain reserve.25 In the biological sense of brain reserve, the brain in the stroke survivors likely sustains a certain level of stroke injury before clinical or cognitive deficit emerges.5,26 However, cognitive reserve may also play a role in sustaining the severity of injury, and*	Page 13 line 17

		the degree of cumulative lesion burden as factors in cognitive outcomes"	
9	Your paper would be much enhanced if brain morphometry measures/ mediating factors where included. In case such data are available, it is definitely worth trying exploring this further	This is a good point. We have neuroimaging morphometric assessments only on a third of the sample. Previously we showed that both global and medial temporal lobe atrophy were predictive of dementia. Although numbers of those who had imaging are limited in the 12-year data set we think it will require additional thorough analyses, which we feel will complicate the findings being reported here and are beyond the scope of this report.	
Reviewer			
1	This manuscript reports a prospective study of cognitive function and dementia incidence after stroke. The findings are potentially interesting, as the literature on post-stroke dementia has tended towards an Alzheimer-like picture of gradual decline, with the dementia precipitated but not necessarily caused by the stroke event, whereas this study suggests that the textbook step-wise decline may be more prominent than previously thought. The statistics are relatively complex and (in my view) require specialist evaluation, so my comments below are based on the assumption that the correct interpretations are being made from these	Thank you.	
2	On p6 (Trajectories of cognitive change section), it would be helpful for the reader to have the rationale explained for censoring of the last years of follow-up.	Thank you for highlighting this point. Censuring was selected to formally test if cognitive change follows a continuous or stepwise pattern. This was the most robust analysis possible, in light of the data available and power limitations.<br>In continuous change-over-time, censoring the last years of follow-up should have a minimal effect on the yearly rate of change. The inverse is true for a stepwise pattern. These results are presented in supplemental Table 1 and 2, for CAMCOG and MMSE respectively.<br>The results section now reads: *"The latter analysis, censuring the last three years of cognitive measures investigates whether cognitive change follows a stepwise or continuous pattern. In continuous change over-time censoring last years of follow-up should have a minimal effect on the yearly rate of change, but a marked difference if the pattern is stepwise"*	Page 6 Line 24
3	For the first paragraph of the Results (p8), 'person years' would be more	We agree. This is now corrected	Page 9 line 2

	appropriate than 'years' for the follow-up durations		
4	Results, second paragraph, first line: is 'points per year ...' intended?	Thanks. This is now corrected.	Page 9<br>Line 6
5	It would help to know a bit more about the representativeness of the sample. Is there a participation rate that could be cited? How does the incidence of post-stroke dementia compare with reports from other cohorts?	These are good points. We have previously reported on recruitment and incidence rates (Allan et al, 2011). However, we have now added to the discussion to indicate current UK figures from the OXVASC study.<br>This discussion now includes: *"Our findings on the incidence of dementia of 26% are also generally in agreement with those from the Oxford Vascular Study reporting 34% incidence of post-event dementia at 1 year, particularly in patients with severe stroke (refs.20,21). Interestingly, these incidence rates are not very different from those in Nigerian stroke survivors with different dietary and lifestyle factors."*	Page 12<br>Line 7
6	The interesting divergence from other findings of gradual cognitive decline after stroke would benefit from a bit more discussion. For example, quite a lot of cases of gradual (Alzheimer-like) cognitive decline after stroke may have had pre-stroke decline. Do the authors feel that this might have been screened out more rigorously than in other studies? If so, this might result in a more dominating step-like picture.	This is a good point. Pre-stroke decline is possible, but this was difficult to detect as the majority at baseline were cognitively intact. Our pathophysiological analysis also suggests that the majority of the survivors who developed dementia could be diagnosed with VaD, without sufficient AD like pathology.	
Scientific editor			
1	Please, include in the legends the quantitative parameters for all the plots in the figures	We have now addressed this comment and provided the rates of changes and the p values in the Legends for Figures 1 to 4.	Figure 1<br>Figure 2<br>Figure 3<br>Figure 4
2	-Please supply a graphical abstract. A graphical abstract is a visual representation of the central finding or methodology of the paper. It will be displayed in the table of contents. Authors should strive to make the graphical abstract informative, interesting, visually appealing, and straightforward (no caption needed, no abbreviations except gene names). Please upload as a separate high resolution image file (tiff preferred). The technical requirements for the graphical abstracts are:<br>--size of the submitted image: 1200 pixels square at 300dpi	We have now provided one.	Submitted

	--font of the text: Arial 12-16 point (smaller fonts will not be legible online)		
3	-Please add an Abbreviated Summary (up to 50 words) of your paper that captures the main purpose and conclusions of the work. The summary should state the main results written in the third person ('[1st author surname] et al. report that …') and conclusion. This summary will be used in the contents list online and should not contain any abbreviations other than those on the accepted abbreviations list and accepted gene/protein names, making it accessible to the non-specialist. Please upload it as a Word document. Allowed abbreviations: AIDS; ANOVA; AMPA; ATP; A,T,C,G; CNS; CSF; CT; DNA; ECG; EEG; EMG; GABA; HIV; MRI; NMDA; PET; PCR; RNA.	We have now added an abbreviated summary. "Stroke increases risk of dementia in ~30% of the survivors. Our unique prospective-longitudinal study showed cognitive decline following stroke occurs in two stages; a period of cognitive stability followed by rapid decline ~3 years prior to dementia. We suggest stepwise development of dementia even when patients have small ischemic lesions."	Page 2 Line 14
