# Supplementary material for: Trajectories of cognitive change following stroke: stepwise decline towards dementia in the elderly
Source: Brain Commun. 2022 May 24;4(3):fcac129. doi: 10.1093/braincomms/fcac129 (PMC9161377; doi:10.1093/braincomms/fcac129)
Supplement: fcac129_Supplementary_Data [file Original Submission - Decision Letter.pdf › Original Submission - Decision Letter.pdf]

Original Submission – Decision Letter

10-Dec-2021

Dear Dr. Delgado,

Thank you for your recent submission BRAINCOM-2021-399
entitled "Trajectories of cognitive change following stroke: stepwise decline towards dementia in the elderly" to Brain Communications.

Your manuscript has been reviewed, and while the reviewers had largely positive comments, they have some constructive critiques that I think you could address in a minor revision of the manuscript. Therefore, we invite you to respond to the reviewer(s)' comments and revise your manuscript. The reviewer(s)' comments are included at the bottom of this message.

In addition to the reviewers' comments, given that the statistics for this paper are relatively complex and the results hinge on their correct application and interpretation we recommend as suggested by one of the reviewers that specialist statistician review is sought during the revision of the paper if the authors cannot easily clarify the statistics issues raised. Furthermore, also kindly address our Scientific Editor's comments added below to comply with our requirements for presentation of scientific data at Brain Communications.

Please format your revised manuscript according to our Instructions to Authors https://academic.oup.com/braincomms/pages/General_Instructions – a summary of key points is attached to help with this.

To revise your manuscript, click the link below or log into https://mc.manuscriptcentral.com/braincom and enter your Author Centre, where you will find your manuscript title listed under "Manuscripts with Decisions." Under "Actions," click on "Create a Revision." Your manuscript number will be appended to denote a revision.

*** PLEASE NOTE: This is a two-step process. After clicking on the link, you will be directed to a webpage to confirm. ***

https://mc.manuscriptcentral.com/braincom?URL_MASK=e3ef2858a2ba432aa6fcb01313450076

Please upload a revised manuscript with the changes you have made shown either in coloured text or using "track changes".

When submitting your revised manuscript, you will be asked to respond to the reviewer(s) comments. Please be as specific as possible in your response to all of the comments.

The editorial team at Brain Communications also aims to increase transparency in scientific publishing and to demonstrate the value that peer review brings to the scientific process. Please therefore consider whether you are willing to have reviewer comments on your paper and your response published as supplementary data with the paper, if it is accepted. This is entirely optional and will only be done if both the authors and reviewers agree. When submitting your revision you will have the opportunity to update the answer you entered previously.

IMPORTANT: Your original files are available to you when you upload your revised manuscript. Please delete any redundant files before completing the submission.

Because we are trying to facilitate timely publication of manuscripts submitted to Brain Communications, we would appreciate your revised manuscript as soon as possible. If it is not possible for you to resubmit within 3 months, the option to submit a revision will expire, and we would then consider your revised paper as a new submission. However, if you need a little more time, please get in touch and we can extend this deadline for you.

Once again, thank you for submitting your manuscript to Brain Communications and I look forward to receiving your revision.

Best wishes,

Professor Alfred K. Njamnshi
Associate Editor, Brain Communications

Professor Tara Spires-Jones
Founding Editor, Brain Communications

Reviewer(s)' Comments to Author:

Reviewer: 1 (Angeliki Tsapanou)

Comments to the Author
The current study investigates trajectories of cognitive change/ incident dementia after stroke. This is a well-organized and well written research, adding interesting insights into the field of cognitive aging/ dementia. Big strength the length of the follow-up and the relatively big sample size.
-Did you include MCI at baseline? If no, please add this info in the Methods section, if yes, please clarify and probably report results with/out the MCI group.
-Cognitive outcomes: did you transform to z-scores? Does higher score indicate better performance?
-Why both CAMCOG and MMSE were used? MMSE is quite short and is more of a limitation to make research conclusions regarding cognition based on this.
-Make clear the cognitive domains and the analyses regarding them, both in the Methods and the Results sections.
-Statistical analysis: did you use any covariates? I would recommend at least using age, sex, and education (probably BMI too) (it is only mentioned at the line plot, but which ones where used, in which models etc.? Also, confused with the Risk factors, are they used as a covariate?)
-Results: What type of dementia was seen in the follow-up? Percentages of vascular vs other?
-Discussion: do you think is that a matter of Cognitive Reserve or Brain Reserve? Please advise Stern et al., 2020 (Whitepaper) and expand more.
-Your paper would be much enhanced if brain morphometry measures/ mediating factors where included. In case such data are available, it is definitely worth trying exploring this further.

Reviewer: 2

Comments to the Author
This manuscript reports a prospective study of cognitive function and dementia incidence after stroke. The findings are potentially interesting, as the literature on post-stroke dementia has tended towards an Alzheimer-like picture of gradual decline, with the dementia precipitated but not necessarily caused by the stroke event, whereas this study suggests that the textbook step-wise decline may be more prominent than previously thought. The statistics are relatively complex and (in my view) require specialist evaluation, so my comments below are based on the assumption that the correct interpretations are being made from these:

1. On p6 (Trajectories of cognitive change section), it would be helpful for the reader to have the rationale explained for censoring of the last years of follow-up.

2. For the first paragraph of the Results (p8), 'person years' would be more appropriate than 'years' for the follow-up durations.

3. Results, second paragraph, first line: is 'points per year ...' intended?

4. It would help to know a bit more about the representativeness of the sample. Is there a participation rate that could be cited? How does the incidence of post-stroke dementia compare with reports from other cohorts?

5. The interesting divergence from other findings of gradual cognitive decline after stroke would benefit from a bit more discussion. For example, quite a lot of cases of gradual (Alzheimer-like) cognitive decline after stroke may have had pre-stroke decline. Do the authors feel that this might have been screened out more rigorously than in other studies? If so, this might result in a more dominating step-like picture.

Comments from the Scientific Editor:

-Please, include in the legends the quantitative parameters for all the plots in the figures.

-Please supply a graphical abstract. A graphical abstract is a visual representation of the central finding or methodology of the paper. It will be displayed in the table of contents. Authors should strive to make

the graphical abstract informative, interesting, visually appealing, and straightforward (no caption needed, no abbreviations except gene names). Please upload as a separate high resolution image file (tiff preferred). The technical requirements for the graphical abstracts are:

--size of the submitted image: 1200 pixels square at 300dpi

--font of the text: Arial 12-16 point (smaller fonts will not be legible online)

-Please add an Abbreviated Summary (up to 50 words) of your paper that captures the main purpose and conclusions of the work. The summary should state the main results written in the third person ('[1st author surname] et al. report that …') and conclusion. This summary will be used in the contents list online and should not contain any abbreviations other than those on the accepted abbreviations list and accepted gene/protein names, making it accessible to the non-specialist. Please upload it as a Word document. Allowed abbreviations: AIDS; ANOVA; AMPA; ATP; A,T,C,G; CNS; CSF; CT; DNA; ECG; EEG; EMG; GABA; HIV; MRI; NMDA; PET; PCR; RNA.
